# Increased seminal root number associated with domestication improves nitrogen and phosphorus acquisition in maize seedlings

**DOI:** 10.1093/aob/mcab074

**Published:** 2021-06-12

**Authors:** Alden C Perkins, Jonathan P Lynch

**Affiliations:** Department of Plant Science, The Pennsylvania State University, University Park, PA, USA

**Keywords:** Domestication, roots, seed reserves, abiotic stress, nitrogen, phosphorus, *Zea mays* ssp. *mays*, *Zea mays* ssp. *parviglumis*

## Abstract

**Background and Aims:**

Domesticated maize (*Zea mays* ssp. *mays*) generally forms between two and six seminal roots, while its wild ancestor, Mexican annual teosinte (*Zea mays* ssp. *parviglumis*), typically lacks seminal roots. Maize also produces larger seeds than teosinte, and it generally has higher growth rates as a seedling. Maize was originally domesticated in the tropical soils of southern Mexico, but it was later brought to the Mexican highlands before spreading to other parts of the continent, where it experienced different soil resource constraints. The aims of this study were to understand the impacts of increased seminal root number on seedling nitrogen and phosphorus acquisition and to model how differences in maize and teosinte phenotypes might have contributed to increased seminal root number in domesticated maize.

**Methods:**

Seedling root system architectural models of a teosinte accession and a maize landrace were constructed by parameterizing the functional–structural plant model OpenSimRoot using plants grown in mesocosms. Seedling growth was simulated in a low-phosphorus environment, multiple low-nitrogen environments, and at variable planting densities. Models were also constructed to combine individual components of the maize and teosinte phenotypes.

**Key Results:**

Seminal roots contributed ~35 % of the nitrogen and phosphorus acquired by maize landrace seedlings in the first 25 d after planting. Increased seminal root number improved plant nitrogen acquisition under low-nitrogen environments with varying precipitation patterns, fertilization rates, soil textures and planting densities. Models suggested that the optimal number of seminal roots for nutrient acquisition in teosinte is constrained by its limited seed carbohydrate reserves.

**Conclusions:**

Seminal roots can improve the acquisition of both nitrogen and phosphorus in maize seedlings, and the increase in seed size associated with maize domestication may have facilitated increased seminal root number.

## INTRODUCTION

The collection of phenotypic traits that differentiate cultivated plants from their wild relatives is referred to as domestication syndrome (Hammer, 1984). Common domestication traits in plants include the loss of seed dispersal mechanisms, increased size of seeds or fruits, decreased photoperiod sensitivity and changes in secondary metabolite production (Doebley *et al.*, 2006; Meyer *et al.*, 2012). While the transition from natural to agricultural ecosystems is associated with many changes in the rhizosphere, such as supplementary irrigation, the application of chemical fertilizers, control of edaphic pests and diseases and decreased interspecific competition (Schmidt *et al.*, 2016), little is known about how domestication influences root phenotypes.

Maize (*Zea mays* ssp. *mays*) was domesticated ~9000 years ago from Mexican annual teosinte (*Z. mays* ssp. *parviglumis*), and the crop has several major morphological differences from its progenitor (Matsuoka *et al.*, 2002). Teosintes may form many tillers and lateral branches that terminate in male inflorescences, while maize does not typically branch or tiller. The female inflorescences of teosinte are small and have two rows of seeds, while domesticated maize forms large ears with many ranks of seeds (Doebley *et al.*, 1990). Teosinte kernels are small and covered by hard cupulate fruitcases that may constitute half the dry weight of the seed, while maize seeds are exposed and may be ten times larger (Doebley, 2004). Maize landrace seeds contain ~58 % less protein and 34 % more carbohydrates than teosinte kernels as a percentage of kernel mass (Flint-Garcia *et al.*, 2009). The maize embryo contains ~26 % of the seed protein, which is in the form of globulins, and the remaining protein is found in the endosperm (Watson, 2003; Flint-Garcia, 2017). Most of the endosperm protein is in the form of ethanol-soluble proteins called zeins, which constitute a greater percentage of the total seed protein in teosinte than maize (Paulis and Wall, 1977).

The maize root system is composed of the primary root, a variable number of seminal roots, and shoot-borne nodal roots that form in whorls initiated both below ground (crown roots) and above ground (brace roots) (Kiesselbach, 1949) as well as lateral roots on all axial root classes. Seminal root primordia are initiated around the scutellar node in the seed starting ~25 d after pollination (Erdelska and Vidovencova, 1993). They are sometimes classified as dorsal, ventral and intermediary based on their orientation to the embryo axis, and their development begins in approximately that order. Seminal roots emerge from the seed following the primary root, often before the coleoptile has emerged from the soil, and the embryonic roots comprise the majority of the root system for the first 2–3 weeks as the adventitious roots are developing (Feldman, 1994; Hochholdinger *et al.*, 2004). Maize seminal roots are of smaller diameter than the primary root and crown roots, and they have a smaller stele, fewer cortical cell files and fewer metaxylem vessels (Burton *et al.*, 2013*b*; Tai *et al*., 2016).

Variation in seminal root number is found within maize and other monocot species, and domestication appears to have increased the average seminal root number in some species. Burton *et al.* (2013*a*) characterized root anatomical and architectural diversity in a panel of 195 maize landraces and 61 teosinte accessions from diverse geographic origins. They observed that maize landraces had 3·9 seminal roots on average, while teosinte accessions had 0·5 seminal roots on average. The opposite pattern was observed for crown roots; landraces had 20·6 crown roots on average, while teosinte accessions had 24·0 at 28 d after planting. Increased seminal root number following domestication has also been reported in other Poales, such as barley (*Hordeum vulgare*) (Grando and Ceccarelli, 1995). In wheat (*Triticum* spp.), five seminal root primordia are found in the seeds of both cultivars and wild relatives (Robertson *et al.*, 1979), and all five primordia typically develop in cultivated wheat, while only three develop in wild accessions under normal conditions (Golan *et al.*, 2018). Other grains, such as rice (*Oryza sativa*) and sorghum (*Sorghum bicolor*), form a primary root but no seminal roots (Hochholdinger *et al.*, 2004; Singh *et al.*, 2010). Sorghum is a close relative of maize; the two lineages diverged ~12 million years ago (Salvi, 2017). Molecular evidence suggests that many of the genes involved in the formation of maize seminal root primordia may be non-syntenic to sorghum (Tai *et al.*, 2017).

Teosinte and early maize landraces grew in soils with limited nutrient availability. The domestication of maize took place in the lowlands of southern Mexico, a region with tropical deciduous forests, wet summers and dry winters (Hastorf, 2009). Highly weathered soils with low phosphorus availability are found in this region (Krasilnikov *et al.*, 2013), and high levels of precipitation contribute to nitrate leaching. Early maize was brought to the Mexican highlands, where it diversified before spreading to the north and south (Matsuoka *et al.*, 2002). In the highlands, it probably experienced lower temperatures (Eagles and Lothrop, 1994) and low phosphorus due to the presence of Andisols, which have high phosphorus fixation (Bayuelo-Jiménez *et al.*, 2011; Bayuelo-Jiménez and Ochoa-Cadavid, 2014). In other parts of the world, including the central USA, low soil nitrogen may have been a more significant limitation to maize growth (Schlegel and Havlin, 1995), especially before the widespread use of synthetic fertilizers in the 1940s.

Root architecture has implications for soil resource acquisition (Lynch, 1995, 2019). While phosphorus is highly immobile in soils, nitrate, the dominant form of inorganic nitrogen in most agroecosystems, is prone to leaching (Barber, 1995; Di and Cameron, 2002; Kabala *et al.*, 2017). Nitrogen leaching is affected by soil texture and precipitation levels, among other factors (Gaines and Gaines, 1994; Dodd *et al.*, 2000). In the absence of rainfall, soil water may also become relatively more available at depth due to evaporation at the soil surface as well greater root activity in shallow soil (Asbjornsen *et al.*, 2008). Therefore, while maize root angles become steeper relative to the soil surface under low-nitrogen conditions (Trachsel *et al.*, 2013), the ideal root angle for nitrogen capture depends on the environment (Dathe *et al.*, 2016). Trade-offs exist between root architectures that improve the acquisition of phosphorus and those useful for mobile resources like nitrates and water (Ho *et al.*, 2005; Rangarajan *et al.*, 2018). For example, low crown root number is useful for the acquisition of nitrogen (Saengwilai *et al.*, 2014) and water (Gao and Lynch, 2016), but high crown root number improves phosphorus capture (Sun *et al.*, 2018) in maize when these resources are limited. Similarly, low lateral root branching density improves maize growth with low nitrogen (Zhan and Lynch, 2015) and water (Zhan *et al.*, 2015), but high lateral root branching density improves phosphorus acquisition (Jia *et al.*, 2018).

While increased seminal root number improves phosphorus capture (Zhu *et al.*, 2006), it is unclear how the increase in seminal root number associated with domestication influences seedling nitrogen acquisition. At the beginning of the growing season, when seminal roots are likely to be the most important, nitrogen concentrations may be greater in the topsoil if inorganic nitrogen fertilizer has recently been applied (Jobbágy and Jackson, 2001). In the Corn Belt region of the USA, nitrogen mineralization from organic matter may also be greater in the topsoil at the time of planting due to organic matter and soil temperature gradients (Gupta *et al.*, 1982). Therefore, the distributions of nitrogen and phosphorus in the soil profile may be similar during early growth. The ‘steep, cheap and deep’ maize root ideotype proposes two phenotypes for seminal roots in order to complement the nodal roots (Lynch, 2013). If the first crown roots are slow to develop, seminal roots should grow at shallow angles relative to the soil surface and be highly branched in order to capture shallow soil resources. If the first crown roots develop quickly and are sufficient for capturing shallow soil resources, seminal roots should grow at a steeper angle and have less lateral branching in order to capture deep resources. Therefore, it is possible that seminal roots perform multiple functions for domesticated maize.

Functional–structural plant models, which combine 3-D representations of plant architecture with physiological models, can provide useful insights into systems that are challenging to study empirically (Vos *et al.*, 2010; Dunbabin *et al.*, 2013). Understanding the influences of domestication on seminal root number in maize requires the consideration of plant performance in diverse environments and phenotypes intermediate to those of maize and teosinte. Since maize and teosinte differ in several respects, including vigour, tillering and growth habit, it is challenging to understand how individual components of their phenotypes contribute to stress adaptation. Simulation modelling can be a useful approach to understanding maize and teosinte root architectures because it allows traits to be experimentally modified in isolation while other components of the phenotype remain constant. The functional–structural plant model OpenSimRoot includes a detailed root architectural model that accounts for root construction costs, respiration and nutrient uptake at the level of individual root segments (Postma *et al.*, 2017). It also allows for the simulation of low-phosphorus soils and for soil nitrate leaching and depletion to be simulated in three dimensions.

This study aims to understand the impact of maize seminal roots on seedling nitrogen acquisition and examine how maize and teosinte phenotypes might have caused domesticated maize to have a greater number of seminal roots than its wild ancestor. We propose that increased seminal root number improves nitrogen acquisition by maize seedlings, and the large increase in seed size associated with domestication might have facilitated an increase in seminal root number by providing additional resources for growth during the early development of the plant.

## MATERIALS AND METHODS

The functional–structural plant model OpenSimRoot (Postma *et al.*, 2017) was used to evaluate the impacts of seminal root number and other components of maize and teosinte phenotypes on nitrogen and phosphorus acquisition. Model version 2e4779cf was used, which is publicly available (https://gitlab.com/rootmodels/OpenSimRoot). OpenSimRoot uses the SWMS_3D model of water and solute movement (Šimunek *et al.*, 1995) to simulate nitrate leaching with a finite element approach. Organic matter mineralization follows the Yang and Janssen model (Yang and Janssen, 2000). Ammoniacal nitrogen is not modelled, as nitrification is generally rapid in the field conditions being simulated (Barber, 1995). Phosphorus uptake is simulated using the Barber–Cushman model (Barber and Cushman, 1981). Roots are composed of connected cylinders or truncated cones that are generally <1 cm in length. Root segment construction requires carbon and nutrients, and respiration and nutrient uptake are calculated at the root segment level. The optimal nutrient content of the plant is determined based on the size and optimal nutrient concentrations of its different tissue types. Root cortical aerenchyma formation occurs after root construction, and it results in decreased respiration rates and the remobilization of nutrients (Postma and Lynch, 2011*a*, *b*). Distinct root classes are simulated that have individual branching angles, diameters and growth rates, among other things. Shoots are simulated non-geometrically, but a leaf area parameter is included from which light interception and photosynthesis are calculated. Nutrient stresses have defined impacts on the rate of leaf area expansion, photosynthesis and root growth rates. Carbon is partitioned among roots, shoots and leaves according to allocation coefficients.

The existing OpenSimRoot maize model uses plant parameters that are based on empirical measurements, and some of the parameters include stochasticity (Postma and Lynch, 2011*a*). The existing ‘Rocksprings’ and ‘WageningseBovenBuurt’ low-nitrogen environments were used in this study as the silt-loam and sand-textured soils, respectively (described in Dathe *et al.*, 2016). The ‘Rocksprings’ environment is parameterized to resemble the Russell E. Larson Agricultural Research Center in Rock Springs, PA, USA, and the ‘WageningeseBovenBuurt’ environment is based on the ‘De Bovenbuurt’ research field at Wageningen University, the Netherlands. Bulk density and parameters related to soil water retention are different for the two soils, while the same precipitation data and initial concentrations of nitrate by depth, which were measured in Rock Springs in 2009, were used for both environments. Models begin with nitrate being concentrated close to the soil surface, and nitrate leaching and depletion take place over time.

### Model parameterization

Seeds of PI 213706 (*Zea mays* ssp. *mays*) and Ames 21803 (*Zea mays* ssp. *parviglumis*) were acquired from the United States Department of Agriculture (USDA) North Central Regional Plant Introduction Station, Ames, IA, USA. Ames 21803 was collected in the Mexican state of Guerrero, and its root architecture, anatomy and vigour are fairly representative of other *parviglumis* accessions (Burton *et al.*, 2013*a*). PI 213706 is an open-pollinated Midwestern dent landrace sold commercially in the 1920s as Osterland Reid’s Yellow Dent. It is a strain of Reid’s Yellow Dent, which was the most popular cultivar in the Corn Belt region of the USA around the beginning of the 20th century (Troyer, 1999). Osterland Reid’s Yellow Dent is also the parent of many notable maize inbred lines and contributes ~15 % of the background of US maize hybrids (Troyer, 2004). Average seed mass was determined for both genotypes, and the fruitcase was removed from ten representative teosinte seeds to determine the average kernel weight.

To measure parameters relating to plant growth in non-stressed conditions, plants were grown in a growth chamber (Environmental Growth Chambers, Chargin Falls, OH, USA) for 14-h days with 27 ºC day and 17 ºC night temperatures. Approximately 600 µmol m^−2^ s^−1^ photosynthetically active radiation was provided by metal halide bulbs with a photoperiod of 14 h. Seedlings were grown in 1.2 × 0·15 m circular polyvinyl chloride mesocosms lined with plastic. The growing media consisted of 50 % commercial sand (US Silica, Berkley Springs, WV, USA), 30 % vermiculite, 5 % perlite, 15 % field soil (Hagertown silt loam, mesic typic Hapludalf) collected from the Russell E. Larson Agricultural Research Center, and 60 g Osmocote Plus Fertilizer (Scotts Miracle-Gro Company, Marysville, OH, USA). Seeds were planted at a depth of 3 cm and irrigated with tap water as needed.

Four plants of each genotype were destructively harvested 5, 8, 10, 15, 20 and 25 d after planting. Leaf area was measured using a LI-3100 Area Meter (LI-COR Biosciences, Lincoln, NE, USA). Basal segments of primary, seminal and nodal roots were excised for respiration measurements, and lateral roots were removed from these segments using a razor blade. Root respiration was measured using a LI-6400 Portable Photosynthesis System (LI-COR Biosciences) and a custom root chamber by recording carbon dioxide evolution every 5 s for 180 s. Respiration measurements were taken in a climate-controlled room at 22 ºC. Apical and basal root segments preserved in 75 % ethanol were sectioned using laser ablation tomography (Hall et al., 2019; Strock *et al.*, 2019), and root diameter was determined from the resulting images using the ObjectJ plugin for ImageJ (Schneider *et al.*, 2012). Root angle for each whorl was measured on separate plants grown in 10-L pots with three replications. Mesocotyl diameter and growth rate were also measured in separate plants that were grown in tree pots 25·4 cm in height and 6·35 cm in diameter (Stuewe & Sons, Tangent, OR, USA) by destructively harvesting four seedlings every day for 6 d. Existing OpenSimRoot maize parameters were modified to include the measured root angle for each whorl, branching frequency for each root class, seminal root number, nodal root number per whorl, axial root growth rate for each root class, leaf area expansion rate, mesocotyl growth rate and diameter, root respiration rate, root diameter for each root class, time of root emergence for each whorl of nodal roots, and kernel size for both subspecies. Branching frequency values are drawn randomly from a uniform distribution in the existing OpenSimRoot maize model, so a distribution of the same range was centred around the average measured branching frequency value for each subspecies. Growth rates were calculated by fitting second-order polynomial regressions to the measured data and taking the first derivative. At 25 d after planting, teosinte seedlings had two small tillers at most and no tiller roots, so tiller leaf area was considered to be part of the main plant, and tiller formation was not simulated explicitly.

Parameters representing the amounts of seed nitrogen and phosphorus available for seedling growth are included in OpenSimRoot. Rather than measuring total seed nitrogen and phosphorus, which might include nutrients contained in structural compounds that cannot be remobilized, tissue nutrient content was measured on seedlings that were grown for 3 weeks in germination paper (Anchor Paper, St Paul, MN, USA) partially submerged in 5 mm calcium sulphate solution. At the time of sampling, seedlings exhibited nutrient deficiency symptoms and it was assumed that seed nitrogen and phosphorus reserves had been exhausted. For nitrogen content, root and shoot tissue was collected from six seedlings per subspecies, and the tissue was dried, ground and homogenized. Nitrogen was quantified in ~2-mg subsamples using a CHN elemental analyser (EA2400, PerkinElmer, Waltham, MA, USA). For phosphorus content, four samples per subspecies of ~60 mg were digested with nitric acid, and phosphorus was measured using inductively coupled plasma optical emission spectrometry (Avio 200, PerkinElmer). Because of the larger tissue mass required for phosphorus analysis and limited seed availability, *parviglumis* seeds from multiple sources were used for this measurement.

OpenSimRoot includes multiple functions that can be used to simulate the process by which seed carbohydrate reserves become available to the growing seedling. The function commonly used by the maize model calculates seed carbohydrate reserves as a proportion of seed mass and provides those reserves to the developing seedling as demanded by carbon sinks. To account for the changes associated with maize domestication, the carbon module was modified to add a seed carbohydrate content parameter (code available at https://gitlab.com/AP2003/opensimroot-seed-carbohydrate-content). Values for the average kernel carbohydrate content of *parviglumis* accessions and maize landraces reported in Flint-Garcia *et al.* (2009) were used.

Other model parameters were thought to be fairly conserved through domestication or of less importance in 25-d-old seedlings than older plants. Since seminal roots were not observed frequently enough in teosinte for parameterization, the maize seminal root parameters were used in the teosinte model for simulations involving the potential utility of seminal roots to teosinte. Some differences between maize and teosinte are shown in Supplementary Data Table S1, and full parameterization files are provided in Supplementary Data Information S1.

### Simulated environments

Except when stated otherwise, models simulated a single maize plant grown in a monoculture at the ‘Rocksprings’ environment by simulating a rectangular prism of soil 60 cm in width, 26 cm in length and 150 cm in depth, and roots that touched the vertical edges of the simulated area were reflected back in order to account for roots of neighbouring plants. Different rates of available soil nitrogen were obtained by multiplying the initial measured concentrations of nitrate and organic matter at each soil depth by a constant as described by Dathe *et al.* (2016). Phosphorus rates were adjusted by changing phosphorus concentrations by depth in the same way. Planting density treatments were created by maintaining commercial row spacing of 76·2 cm and changing the in-row spacing.

### Optimization

To examine the relationship between seed carbohydrate reserves and the optimal seminal root number for growth under certain low-nitrogen and low-phosphorus conditions, maize landrace plants were simulated with every combination of zero to eight seminal roots and between 10 and 150 mg seed mass, increasing in 2.5-mg increments, with 20 replications per treatment. Seed carbohydrate content as a percentage of seed mass was held constant at 71·12 % [the average value reported for maize landraces by Flint-Garcia *et al.* (2009)], and seed nitrogen and phosphorus reserves were fixed at the values measured for the landrace. The optimal seminal root number for each environment was determined by finding the treatment that maximized shoot dry weight at 25 d after planting. When multiple treatments had the same shoot dry weight after rounding to six decimal places, the treatment with the lowest seminal root number was considered to be optimal in that environment.

### Data analysis

Data were analysed using R version 3.6.2 (R Core Team, 2019) using the packages ‘Biobase’ (Huber *et al.*, 2015) and ‘tidyverse’ (Wickham *et al.*, 2019). Spatial data were processed with Paraview 5.5.2 (Ahrens *et al.*, 2005).

### Statistics

Several OpenSimRoot model parameters are sampled from distributions using a random number generator, which causes stochasticity in model runs. At least six replications with different random number generator seeds were performed for all treatments, and raw data are shown as appropriate to demonstrate when treatment effects are larger than variation due to stochasticity. Where the figure format precludes the display of raw data, averages of at least six replications are shown. Performing statistical hypothesis testing on model results of this type is not appropriate for several reasons: sample size and statistical power are somewhat arbitrary in simulation modelling, which increases the risk of identifying differences that are statistically but not biologically significant; the null hypothesis of no treatment differences is known to be false, which makes the hypothesis framework inappropriate; and structural–functional plant models make certain simplifying assumptions out of necessity, which could mean that the variance present in simulation results is different from what would be observed in a field study (White *et al.*, 2014; Postma *et al.*, 2014). Coefficient of determination values from regression should be interpreted in the context of the simulation assumptions.

### Recombinant inbred line experiment

To examine the relationship between seed mass and seminal root number in maize inbred lines, seeds of 60 members of a B73 × Mo17 recombinant inbred line (RIL) population (Kaeppler *et al*., 2000) and 95 members of an Ny821 × H99 RIL population (Johnson *et al.*, 2016) were acquired from Dr Shawn Kaeppler (University of Wisconsin, Madison, WI, USA). Average kernel mass was determined based on ten seeds, and three seeds per genotype were germinated and transferred to pouches consisting of germination paper (Anchor Paper) covered with black polyethylene and suspended in nutrient solution as described by Hund *et al.* (2009). The nutrient solution consisted of (in µm) 1000 NH_4_NO_3_, 125 MgSO_4_, 3000 KH_2_PO_4_, 500 CaCl_2_, 12·5 H_3_BO_3_, 1 MnSO_4_·2H_2_O, 1 ZnSO_4_·7H_2_O, 0·25 CuSO_4_·5H_2_O, 1·5 Na_2_MoO_4_·2H_2_O and 100 Fe-DTPA. The pH was adjusted to 6·0 daily using 3 N KOH. Seedlings were grown in fluorescent light racks with a photoperiod of 14 h and at a temperature of 22 ºC. Seminal root number was recorded 14 d after planting.

### Comparison with other species

To quantify the variation in seed mass and seminal root number that is present in other species, accession records that include seed mass were obtained for all landraces of barley, oat (*Avena sativa*), pearl millet (*Cenchrus americanus*), rice, rye (*Secale cereale*), sorghum and wheat that are available from the USDA National Plant Germplasm System. Values for the variation in the number of seminal roots present in each species were obtained from the literature. References for each species can be found in Supplementary Data Table S2.

## RESULTS

### Spatiotemporal nature of nutrient acquisition by seminal roots in domesticated maize

**Fig. 1.**
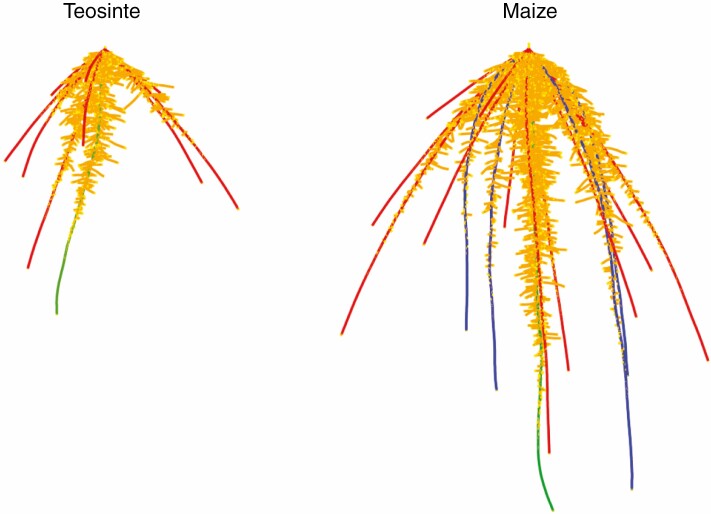
Graphical renderings of the teosinte and maize root system models at 25 d after planting when grown without nutrient stress. Roots are dilated for clarity. Root classes are coloured as follows: green, primary root; blue, seminal roots.

**Fig. 2.**
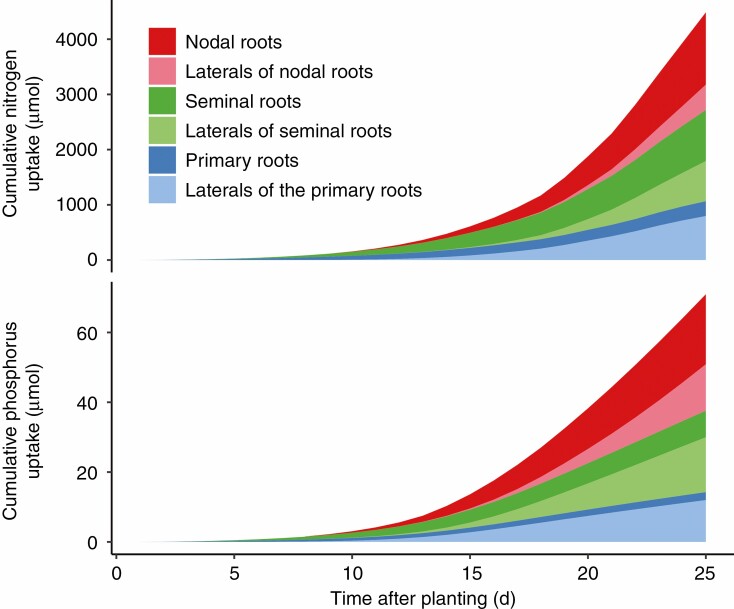
Cumulative contribution of each root class of a simulated maize landrace plant to nutrient acquisition in field conditions with 50 kg ha^−1^ available nitrate (top) and 2 kg ha^−1^ available phosphorus (bottom). These levels of nutrient availability lead to ~50 % reduction in shoot dry weight at 25 d after planting. The values presented are averages from six model replications that include stochasticity. Nodal roots emerged 7 d after planting.

**Fig. 3.**
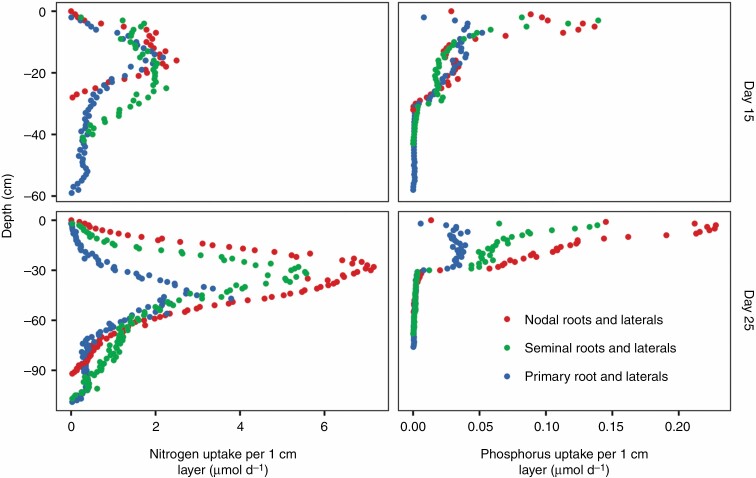
Contributions of each root class of a maize landrace to the acquisition of nitrogen and phosphorus by depth on the 15th and 25th days following planting in field conditions with 50 kg ha^−1^ available nitrate (left) and 2 kg ha^−1^ available phosphorus (right). Root segments are binned by 1-cm soil layers, and the values presented are averages from six model runs that include stochasticity.

Maize landrace models had larger and deeper root systems than those of teosinte at 25 d after planting (Fig. 1). Up to 25 d after planting, seminal roots and their laterals were responsible for ~35 % of the nitrogen and phosphorus acquired by maize in field conditions with 50 kg ha^−1^ available nitrate and 2 kg ha^−1^ available phosphorus (Fig. 2). Lateral roots acquired more phosphorus than axial roots, while axial roots acquired more nitrogen than their laterals during the first 25 d of growth. Nitrogen and phosphorus capture by nodal roots is likely to overtake that by seminal roots after that time. Because seminal roots emerged before nodal roots, they were able to acquire nitrogen from deeper in the soil profile than nodal roots at 15 d after planting (Fig. 3). By 25 d after planting, the contributions of seminal and nodal roots to nutrient acquisition by depth were more similar. In teosinte, nodal roots acquired a larger percentage of the total nitrogen and phosphorus than they did in maize (Supplementary Data Fig. S1), and, like maize, the primary root was able to acquire deeper nitrogen than the nodal root system at 15 d after planting (Supplementary Data Fig. S2).

### Utility of seminal roots to domesticated maize in different environments

**Fig. 4.**
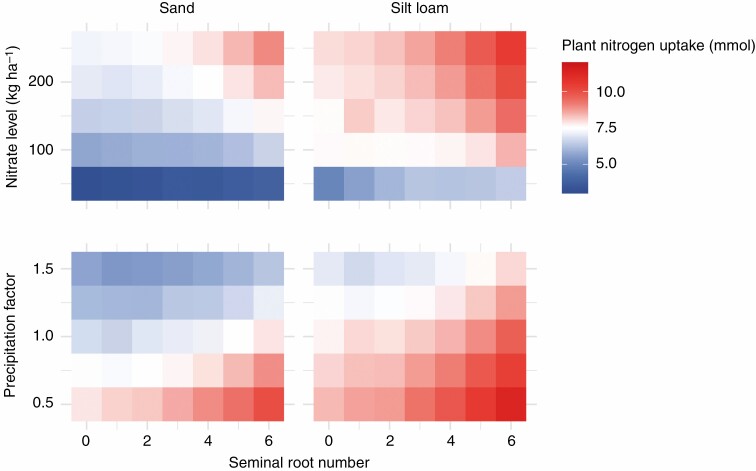
Nitrogen acquisition of a maize landrace with varied seminal root number at 25 d after planting in environments with different initial nitrate concentrations, precipitation regimes and soil textures. Precipitation treatments were created by multiplying the measured daily rainfall by a constant. The values presented are averages of replicated model runs that include stochasticity.

**Fig. 5.**
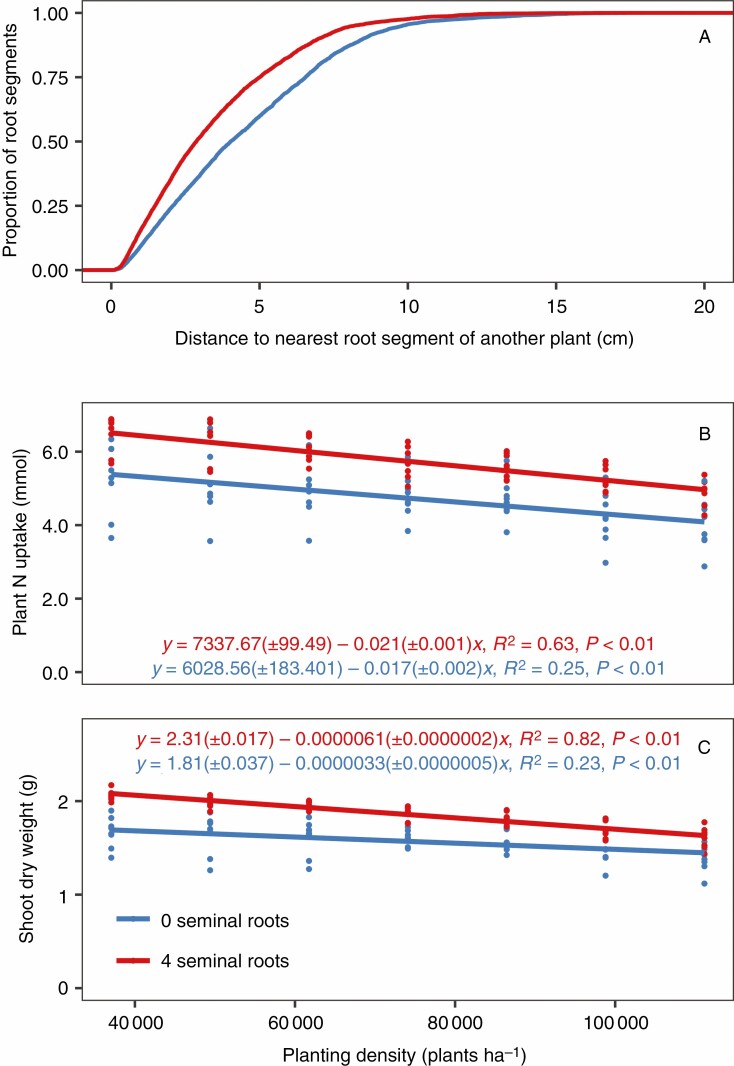
Impact of planting density on the utility of seminal roots. (A) Cumulative distribution functions of the distance from a randomly selected root segment of a maize landrace to the nearest root segment of a neighbouring plant at a planting density of 74 100 plants ha^−1^ (close to commercial planting density) at 25 d after planting. Root position data from six model runs per treatment were subsampled. Nutrient stress is not included in order to avoid confounding allometric effects. On average, the nearest root of a neighbouring plant is closer at 25 d after planting if seminal roots are formed. (B) Nitrogen (N) acquisition and shoot dry weight of a maize landrace at 25 d after planting in a soil with 50 kg ha^−1^ available nitrate when planting density is varied. Points represent values from individual model runs.

**Fig. 6.**
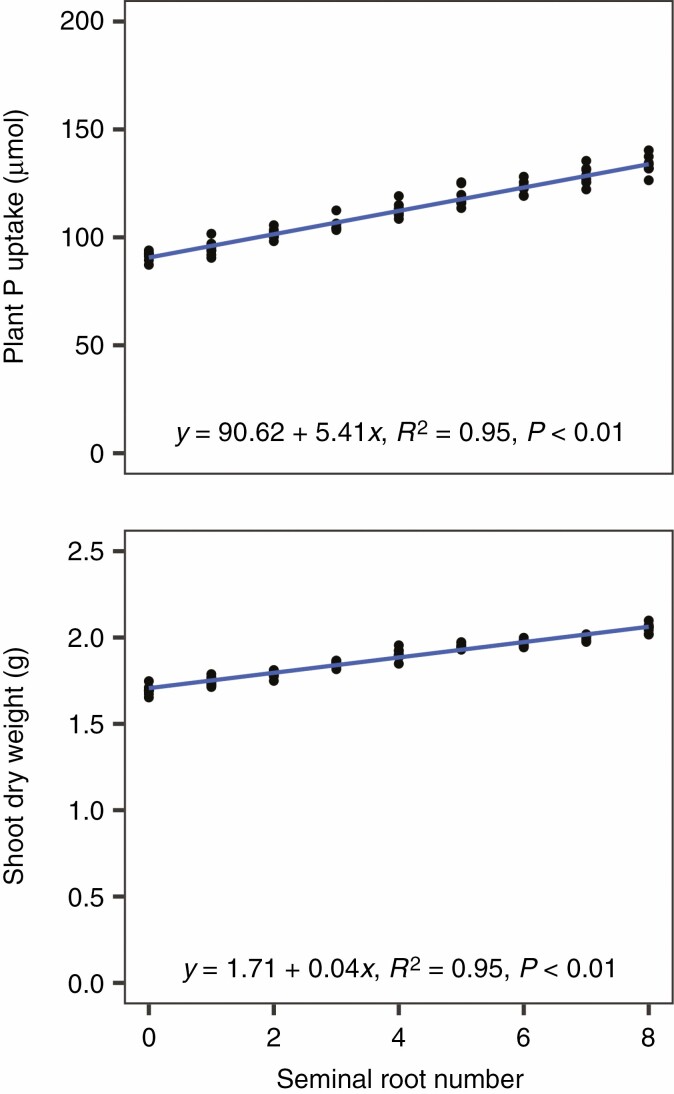
Impact of seminal root number on phosphorus (P) acquisition (top) and shoot biomass (bottom) of a maize landrace in a soil with 2 kg ha^−1^ available phosphorus at 25 d after planting. Points represent values from individual model runs.

Since nitrate is highly mobile in the soil, its location over the course of a growing season depends on precipitation and soil texture, among other factors (Gaines and Gaines, 1994; Dodd *et al.*, 2000). Therefore, the utility of root phenotypes for nitrate capture may depend on the environment (Dathe *et al.* 2016). However, seminal roots were beneficial for nitrogen acquisition in soils of two different textures with all the precipitation regimes and nitrate fertilization rates that were simulated (Fig. 4). Precipitation was inversely related to plant nitrogen acquisition. The presence of seminal roots decreased the average distance between root segments of neighbouring plants (Fig. 5), which might have implications for interplant competition. However, increasing planting density from 37 050 to 111 150 plants ha^−1^ reduced plant nitrogen acquisition by <1 mmol in the first 25 d after planting because little interplant competition for nitrogen occurred during this period. Seminal roots improved nitrogen acquisition and seedling growth for all planting density treatments simulated. Increased seminal root number also improved phosphorus acquisition and plant growth in a simulated low-phosphorus environment (Fig. 6), which has been previously reported by a field study (Zhu *et al.*, 2006).

### Utility of seminal roots to teosinte with low nitrogen and low phosphorus

**Fig. 7.**
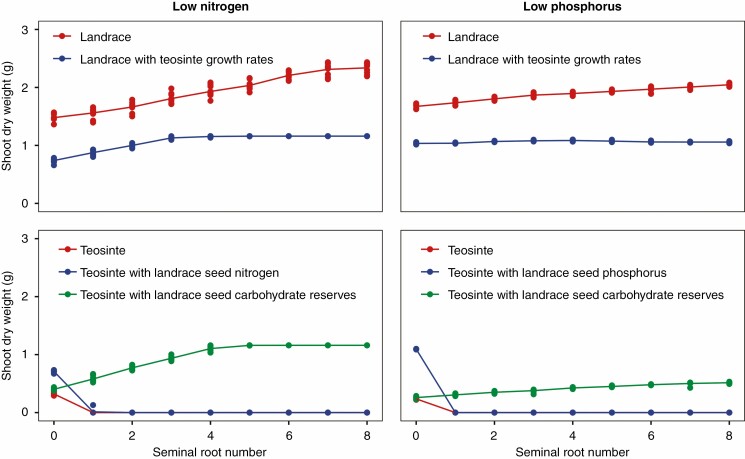
Utility of seminal roots to a maize landrace, teosinte and three composite phenotypes in a low-nitrogen soil with 50 kg ha^−1^ available nitrate (left) and a low-phosphorus soil with 2 kg ha^−1^ available phosphorus (right). Points represent individual model runs, and trend lines pass through the mean value for each treatment. Teosinte treatments with shoot dry weights of ~0 did not have the seed resources necessary for germination.

While increased seminal root number improved the growth of the simulated maize landrace under both low nitrogen and low phosphorus, this effect was less pronounced when the landrace was simulated as having the same root and shoot growth rates as the teosinte accession, which grows more slowly and therefore needs less nitrogen and phosphorus at 25 d after planting (Fig. 7). In the low-nitrogen environment, this composite phenotype experienced no benefit from forming more than three seminal roots because sufficient nitrogen had been captured. In the low-phosphorus environment, the landrace simulated as having teosinte growth rates did not benefit from forming any seminal roots. Seminal roots emerge from the seed before the coleoptile has reached the soil surface and when the seedling is still in a heterotrophic state. With the teosinte model, forming two or more seminal roots left teosinte with insufficient seed resources to germinate in both environments. When teosinte was simulated as having the same amounts of seed nitrogen and phosphorus that are available from maize seeds, it was able to grow larger in the low-nutrient environments, but the optimal number of seminal roots was not impacted. When it was simulated as having the carbohydrate reserves that are present in maize seeds, however, the optimal number of seminal roots was increased in both the low-nitrogen and low-phosphorus environments.

**Fig. 8.**
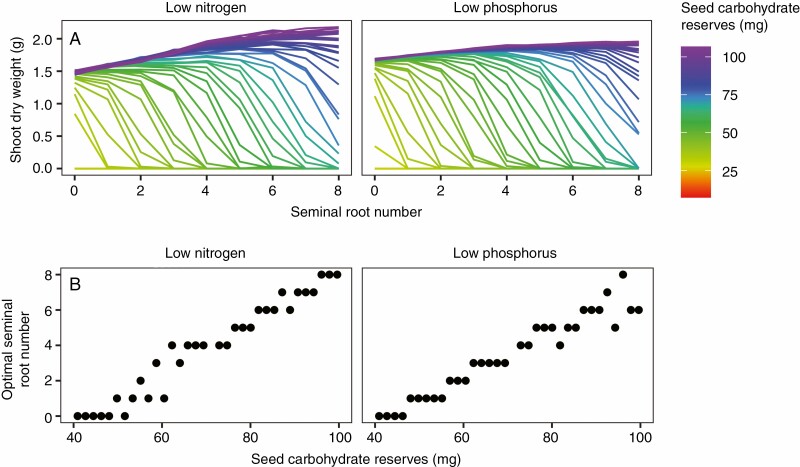
Relationship between seed carbohydrate reserves and optimal seminal root number in the maize landrace model using a low-nitrogen soil with 50 kg ha^−1^ available nitrogen (left) and a low-phosphorus soil with 2 kg ha^−1^ available phosphorus (right). (A) Response curves for each seed carbohydrate reserve treatment. Plotted values are means of 20 replicates for each combination of seminal root number and seed carbohydrate reserves. (B) Optimal seminal root number for each seed carbohydrate content treatment, which was determined from the response curves. When shoot dry weight was equal for multiple seminal root number treatments after rounding to six decimal places, the lower seminal root number was considered to be optimal. Simulations were conducted for a greater range of seed carbohydrate content values than are shown. Seed nitrogen and phosphorus contents were held constant.

Simulations of maize landraces with seed carbohydrate reserves intermediate to those of teosinte and the maize landrace suggest that the optimal seminal root number for plant growth in low-nitrogen and low-phosphorus environments may be dependent on carbohydrate reserves within a certain range of values (Fig. 8). Simulated landraces with <45 mg of seed carbohydrates did not benefit from forming seminal roots in either the low-nitrogen or low-phosphorus environments, while plants simulated with slightly more carbohydrate reserves maximized growth in both environments by forming a limited number of seminal roots. Seminal root number did not appear to be correlated with seed size in two populations of maize recombinant inbred lines that were examined (Supplementary Data Fig. S3). All lines in these populations had seeds of mass >125 mg on average, which translates to ~89 mg of seed carbohydrates if maize seeds are 71·2 % carbohydrates by mass.

As a result of the differences in seed mass and carbohydrate content between maize and teosinte, a much greater proportion of the starting seed carbohydrate reserves were depleted by 7 d after planting in teosinte than in maize (Supplementary Data Fig. S4). Low-nitrogen and low-phosphorus stresses, however, were not experienced until at least 10 d after planting (Supplementary Data Fig. S4). Due to the carbohydrate limitation, trade-offs may also exist between increased seminal root number and the growth of the primary root. In teosinte, the presence of additional seminal roots decreased the average length of the primary root and the length of the longest seminal root at 7 d after planting (Supplementary Data Fig. S5).

### Seminal root number and seed mass in other species

**Fig. 9.**
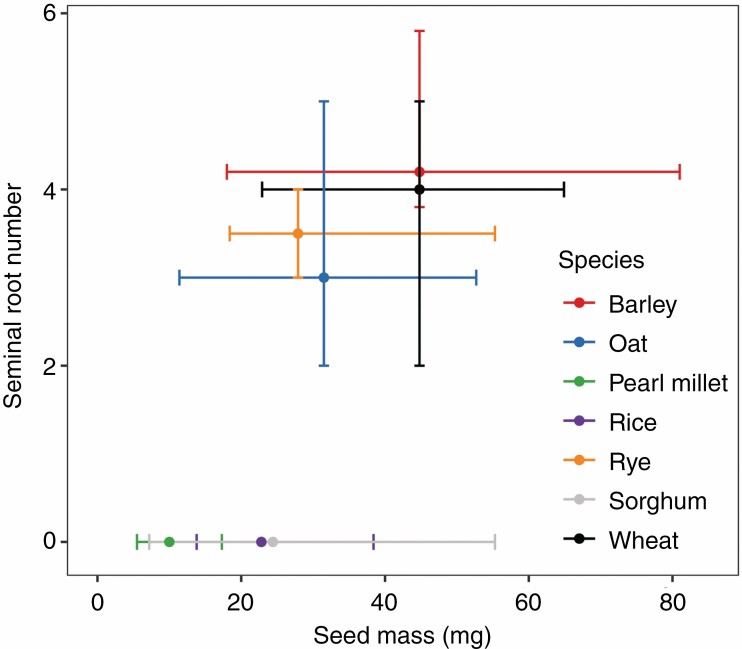
Variation in seminal root number and seed mass in domesticated barley, oat, pearl millet, rice, rye, sorghum and wheat based on the literature and germplasm repository records. Following the definition of seminal roots commonly applied to maize (Hochholdinger *et al.*, 2004), the radicle is not considered a seminal root. The mean, minimum and maximum seed mass values are calculated using all landraces for which data are available from the United States Department of Agriculture National Plant Germplasm System. Sources for seminal root number values can be found in Supplementary Data Table S2.

The average kernel mass of teosinte is ~30 mg while the average kernel mass of maize landraces is ~280 mg, and carbohydrate content as a proportion of seed mass is lower in teosinte than landraces (Flint-Garcia *et al.*, 2009). Germplasm records and data obtained from the literature suggest that some Poales with seeds intermediate in mass to those of maize and teosinte, such as barley, oat, rye and wheat, form a variable number of seminal roots, while other species with seeds of <30 mg on average, such as pearl millet, rice and sorghum, do not form seminal roots (Fig. 9). Differences in growing environments, morphology, seed composition and carbon partitioning, among other things, might impact the number of seminal roots formed by other species. References for seminal root number values in other species can be found in Supplementary Data Table S2.

## DISCUSSION

OpenSimRoot was used to understand the spatiotemporal dynamics of nutrient acquisition by the seminal roots of maize, the utility of seminal roots to maize in different low-nutrient environments, and the degree to which differences in the growth and morphology of maize and teosinte impact the seedling root architectures of each subspecies. The results suggest that seminal roots are beneficial for both nitrogen and phosphorus acquisition during the development of maize seedlings, and seminal roots can improve nitrogen acquisition in environments with several different precipitation regimes, fertilization rates and soil textural classes. High numbers of seminal roots may not be beneficial to teosinte because its lower growth rates mean that it has lower nutrient requirements as a seedling and because its small seeds have smaller carbohydrate reserves to support seedling growth.

The impact of seminal root number on seedling performance under drought was not simulated in this study because drought responses are not yet fully implemented in OpenSimRoot. In the Corn Belt region of the USA, water is often available in the topsoil at the time of planting because of snowmelt and spring rains (Asbjornsen *et al.*, 2008), so seedling water acquisition efficiency may be of little importance. In certain arid regions, such as the Central Rift Valley of Ethiopia, the Mexican highlands and the south-western USA, some indigenous growers have planted maize at depths of 10 cm and greater in order to ensure that sufficient moisture is available for germination (Collins, 1914; Eagles and Lothrop, 1994; Liben *et al.*, 2015). Maize genotypes with reduced crown root number have deeper rooting and greater shoot biomass when grown under drought stress (Gao and Lynch, 2016), so it is possible that a trade-off exists between the ideal seminal root number for nutrient acquisition and water acquisition in certain arid environments.

While OpenSimRoot includes models of nitrogen mineralization, leaching and depletion, it does not currently simulate soil temperature effects on mineralization or root respiration explicitly. In some parts of the world, maize may be planted into cold soils that have decreasing soil temperatures with depth (Gupta *et al.*, 1982). This gradient might impact root maintenance costs or nutrient availability, among other things. Similarly, the model does not include soil hardness effects, which might be important because maize seminal roots are smaller in diameter than primary and nodal roots (Tai *et al*., 2016). Increased root diameter may be associated with improved root penetration in compacted soils (Clark *et al.*, 2003), while thin roots may be more able to grow through small pores (Potocka and Szymanowska-Pulka, 2018). Therefore, the usefulness of seminal roots in compacted soils is unclear.

Some of the comparisons made in this study would be challenging to validate empirically, such as the use of models that combine parts of the maize or teosinte phenotype with the phenotype of the other subspecies. Other components, such as the utility of seminal roots under low-nitrogen stress, could be validated if otherwise similar maize lines with contrasting seminal root number were available. This study uses models that were parameterized to resemble one maize landrace and one teosinte accession, but modelling more of the diversity of both subspecies could also be informative. Teosinte is highly plastic, and teosinte plants that are more mature than those simulated in this experiment respond to suboptimal nitrogen availability by reducing their tiller number (Gaudin *et al.*, 2011). This study considers only the first 25 d of growth because this is the period during which seminal roots make the largest contribution to nutrient acquisition, and tiller roots were not present during this time period in the plants used for parameterization. The study of teosinte *ex situ* is also challenged by its photoperiod requirement (Minow *et al.*, 2018) and sensitivity to inbreeding depression. Finally, seed nutrient content is somewhat variable, and maize grown in low-nitrogen environments forms kernels with decreased protein content (Mayer *et al.*, 2012). Therefore, it is possible that the environment in which seed production occurs could influence some of the model parameters used here.

The relationship between increased seminal root number and improved phosphorus acquisition has previously been reported (Zhu *et al.*, 2006), but the benefit of seminal roots in low-nitrogen soils is a new finding. Trade-offs sometimes exist between nitrogen and phosphorus acquisition because phosphorus is generally concentrated in the topsoil and is fairly immobile (Lynch and Brown, 2001), while nitrate, the dominant form of nitrogen in most agricultural soils, is mobile (Wiersum, 1962). Growers often apply nitrogen fertilizer just prior to planting in order to reduce losses to leaching (Vetsch and Randall, 2004). Therefore, acquisition of both resources may be improved by high seminal root number because both resources are abundant in the topsoil during seedling establishment, while by the time of flowering shallow nitrogen is more likely to have been leached or depleted. Soil organic matter tends to be concentrated in the topsoil (Rethemeyer *et al.*, 2005), so mineralization may contribute to the availability of shallow nitrogen. Ammonium nitrogen is also likely to be concentrated in shallow soil because of its interactions with soil cation exchange sites.

While teosinte probably experiences interspecific competition for resources, modern maize production takes place in high-density, genetically uniform stands. Therefore, interplant root competition might contribute to fitness in the wild, but it may be undesirable in high-input agroecosystems (Denison *et al.*, 2003). Root competition for phosphorus can be quantified by measuring the overlap of depletion zones because phosphorus primarily moves in the soil through diffusion (Rubio *et al.*, 2001), while competition for mobile resources like nitrate and water is more dynamic. Nitrate is primarily acquired by plants through mass flow, and mass flow rates are related to the nutrient concentration in the soil and the rate of water uptake by the plant (Barber, 1995). Increasing planting density had only small impacts on plant nitrogen acquisition at 25 d after planting (Fig. 5), so seminal root number is not likely to have implications for interplant competition in agroecosystems. Some wild environments might have greater plant densities than those simulated here, however.

Seeds of maize landraces are approximately ten times larger than those of teosinte (Flint-Garcia *et al.*, 2009). Small seed size may be adaptive for dispersal and population growth (Moles *et al.*, 2005; Turnbull *et al.*, 2012), while large seeds may be better for seedling establishment and stress tolerance (Wang *et al.*, 2020). The presence of the cupulate fruitcase surrounding teosinte kernels may have also constrained the seed size of the wild ancestors of maize, so selection for naked kernels may have preceded selection for increased seed size (Flint-Garcia, 2017). Seminal roots can emerge from maize seeds before photosynthesis has started and when the primary root is still small, so it may be advantageous for teosinte to apply all of its limited seed carbohydrate reserves to the growth of the radicle and coleoptile, while domesticated maize has more seed carbon, which can support optimal growth of the radicle and coleoptile in addition to seminal root development. It seems unlikely that the formation of seminal root primordia itself is constrained by the physical size of the seed because the primordia are quite small (Taramino *et al.*, 2007).

Growth rates limit the utility of seminal roots to teosinte because it has a smaller nitrogen and phosphorus requirement as a seedling than maize due to its smaller size. The increase in seedling vigour associated with domestication may have occurred for several reasons. Genotypes that produce more secondary compounds to reduce herbivory often have lower growth rates, and herbivory may be a greater challenge in the wild than in managed agroecosystems (Paul-Victor *et al*., 2010; Züst *et al.*, 2011). Genome size appears to be inversely related to leaf elongation rate (Bilinski *et al.*, 2018), and the genome sizes of maize landraces tend to be smaller than those of teosintes, but there is considerable variation (Díez *et al.*, 2013). While growth rates and seed resources are parameters that can be separated in OpenSimRoot, maternal investment may also be biologically related to seedling vigour. Seed mass and seedling size are correlated in several species (Wulff, 1986; Zhang and Maun, 1990; Moegenburg, 1996), so the increased growth rates of maize relative to teosinte may also have been coupled with the increase in seed size. Studies on the impacts of maize seed size on crop growth suggest that large seeds may be associated with increased plant height in seedlings, but seed size is not related to yield or the size of the mature plant in non-stressed conditions (Kiesselbach, 1937; Hunter and Kannenberg, 1972).

Considerable variation in seminal root number is present in maize landraces and inbred lines, and between zero and eight seminal roots are commonly reported (Zhu *et al.*, 2006; Burton *et al.*, 2013*a*). Since most maize seeds are larger than 150 mg, our results suggest that seed nutrient and carbohydrate content should not limit the optimal seminal root number in the domesticate as long as nutrient and carbohydrate contents as a fraction of seed mass are similar to what we simulated. Some types of maize with unusual endosperm properties, such as northern flint corn, sweet corn and popcorn, generally have fewer seminal roots than dent maize lines (Wiggans, 1916; Siemens, 1929), which might be related to their seed composition, lack of vigour relative to dent maize, or other factors. Low seminal root number might also be adaptive in certain types of environments not simulated in this study.

Domesticated wheat and barley form more seminal roots than their wild progenitors, although the average difference in seminal root number between maize and teosinte is larger than the differences observed in these species. Grando and Ceccarelli (1995) screened seminal root number in a small collection of modern barley cultivars, landraces and wild *Hordeum spontaneum* accessions and found that modern cultivars had seeds with an average mass of 49.4 mg and ~4·5 seminal roots on average, while wild *H. spontaneum* seeds were 28·5 mg on average and had an average of ~2·5 seminal roots. Shoot dry weight was also greater on average in modern cultivars than *H. spontaneum*, but there was large variation within each germplasm group. The same number of seminal root primordia is found in the seeds of diploid and tetraploid wild and domesticated wheat species, but more primordia develop in the domesticates (Robertson *et al.*, 1979; Golan *et al.*, 2018). Grain weight is significantly greater in cultivated durum wheat (*Triticum turgidum* ssp. *durum*) than in wild emmer (*T*. *turgidum* ssp. *dicoccoides*), but the difference is smaller than that observed in maize (Golan, 2015). While teosinte seeds overlap in mass with those of some species that frequently form seminal roots, such as wild barley, species differences in seed composition, growth rates and other root traits might also influence the optimal seminal root number. For example, wheat seminal roots may be of smaller diameter than those of domesticated maize (Atwell, 1990; Hund *et al.*, 2004), which would influence the construction and maintenance costs of each root. Sorghum offers an interesting comparison with maize because of the genetic and phenotypic similarity between the two species. Wild sorghum seeds are ~18 mg, while seeds of the domesticate may be as large as 58 mg (Wang *et al.*, 2020). This is considerably smaller than most domesticated maize seeds, and seminal roots have not evolved in sorghum (Singh *et al.*, 2010).

While the importance of seminal roots for nutrient acquisition in maize decreases as the plant matures (Fig. 2), seminal root number may still have important agronomic implications. Nitrogen and phosphorus fertilizers are costly inputs for maize growers, and <60 % of the nitrogen fertilizer applied is commonly recovered by the crop (de Oliveira *et al.*, 2018; Mueller *et al.*, 2019). Improving nitrogen acquisition efficiency at all stages of growth, including the seedling stage, has the potential to reduce the pollution from fertilization and increase yields in low-input systems. In addition, low soil temperatures at the time of spring planting may limit soil microbial processes and nutrient availability, which could make seedling nutrient acquisition efficiency more important. Knowledge of how domestication has influenced root systems may also be useful for *de novo* domestication efforts aimed at developing stress-tolerant crops.

## SUPPLEMENTARY DATA

Supplementary data are available online at https://academic.oup.com/aob and consist of the following. Table S1: means, standard errors and sample size per treatment for selected model parameters and other measurements from the maize and the teosinte accession. Table S2: sources of seminal root number data from other species that are shown in Fig. 9. Figure S1: contribution of the primary and nodal roots to nutrient acquisition in teosinte grown in field conditions with 50 kg ha^−1^ available nitrate and 2 kg ha^−1^ available phosphorus. Figure S2: acquisition of nitrogen and phosphorus by teosinte at 15 and 25 d after planting. Figure S3: seminal root number does not appear to be related to seed mass in dent maize recombinant inbred lines resulting from B73 × Mo17 and Ny821 × H99. Figure S4: temporal nature of seedling carbon and nutrient stress. Figure S5: impact of seminal root number on the length of the primary root and longest seminal root in teosinte at 7 d after planting. Information S1: OpenSimRoot maize landrace and teosinte parameterization.

## Supplementary Material

mcab074_suppl_Supplementary_Materials

mcab074_suppl_Supplementary_Data

## References

[CIT0001] Ahrens J, Geveci B, Law C. 2005. Paraview: an end-user tool for large data visualization. In: Hansen CD, Johnson CR, eds. The visualization handbook. Cambridge: Academic Press, 717–733.

[CIT0002] Asbjornsen H, Shepherd G, Helmers M, Mora G. 2008. Seasonal patterns in depth of water uptake under contrasting annual and perennial systems in the Corn Belt Region of the Midwestern US. Plant and Soil 308: 69–92.

[CIT0003] Atwell BJ. 1990. The effect of soil compaction on wheat during early tillering: I. Growth, development and root structure. New Phytologist 115: 29–35.

[CIT0004] Barber SA. 1995. Soil nutrient bioavailability: a mechanistic approach. Hoboken: John Wiley & Sons.

[CIT0005] Barber SA, Cushman JH. 1981. Nitrogen uptake model for agronomic crops. In: Iskander IK, ed. Modeling wastewater renovation: land treatment. New York: Wiley Interscience, 382–489.

[CIT0006] Bayuelo-Jiménez JS, Ochoa-Cadavid I. 2014. Phosphorus acquisition and internal utilization efficiency among maize landraces from the central Mexican highlands. Field Crops Research 156: 123–134.

[CIT0007] Bayuelo-Jiménez JS, Gallardo-Valdéz M, Pérez-Decelis VA, Magdaleno-Armas L, Ochoa I, Lynch JP. 2011. Genotypic variation for root traits of maize (*Zea mays* L.) from the Purhepecha Plateau under contrasting phosphorus availability. Field Crops Research 121: 350–362.

[CIT0008] Bilinski P, Albert PS, Berg JJ, et al. 2018. Parallel altitudinal clines reveal trends in adaptive evolution of genome size in *Zea mays*. PLoS Genetics 14: e1007162. 29746459 10.1371/journal.pgen.1007162 PMC5944917

[CIT0009] Burton AL, Brown KM, Lynch JP. 2013 *a*. Phenotypic diversity of root anatomical and architectural traits in *Zea species*. Crop Science 53: 1042–1055.

[CIT0010] Burton AL, Lynch JP, Brown KM. 2013 *b*. Spatial distribution and phenotypic variation in root cortical aerenchyma of maize (*Zea mays* L.). Plant and Soil 367: 263–274.

[CIT0011] Clark LJ, Whalley WR, Barraclough PB. 2003. How do roots penetrate strong soil? In: Abe J, ed. Roots: the dynamic interface between plants and the earth. Berlin: Springer Science and Business Media, 93–104.

[CIT0012] Collins GN. 1914. Pueblo Indian maize breeding: varieties specially adapted to arid regions developed by Hopis and Navajos. Journal of Heredity 5: 255–268.

[CIT0013] Dathe A, Postma JA, Postma-Blaauw MB, Lynch JP. 2016. Impact of axial root growth angles on nitrogen acquisition in maize depends on environmental conditions. Annals of Botany 118: 401–414. 27474507 10.1093/aob/mcw112 PMC4998975

[CIT0014] Denison RF, Kiers ET, West SA. 2003. Darwinian agriculture: when can humans find solutions beyond the reach of natural selection? Quarterly Review of Biology 78: 145–168. 10.1086/374951 12825416

[CIT0015] Di HJ, Cameron KC. 2002. Nitrate leaching in temperate agroecosystems: sources, factors and mitigating strategies. Nutrient Cycling in Agroecosystems 64: 237–256.

[CIT0016] Díez CM, Gaut BS, Meca E, et al. 2013. Genome size variation in wild and cultivated maize along altitudinal gradients. New Phytologist 199: 264–276. 10.1111/nph.12247 PMC4119021 23550586

[CIT0017] Dodd MB, Lauenroth WK, Burke IC. 2000. Nitrogen availability through a coarse-textured soil profile in the shortgrass steppe. Soil Science Society of America Journal 64: 391–398.

[CIT0018] Doebley J. 2004. The genetics of maize evolution. Annual Review of Genetics 38: 37–59. 10.1146/annurev.genet.38.072902.092425 15568971

[CIT0019] Doebley J, Stec A, Wendel J, Edwards M. 1990. Genetic and morphological analysis of a maize-teosinte F2 population: implications for the origin of maize. Proceedings of the National Academy of Sciences of the USA 87: 9888–9892. 11607138 10.1073/pnas.87.24.9888 PMC55279

[CIT0020] Doebley JF, Gaut BS, Smith BD. 2006. The molecular genetics of crop domestication. Cell 127: 1309–1321. 17190597 10.1016/j.cell.2006.12.006

[CIT0021] Dunbabin VM, Postma JA, Schnepf A, et al. 2013. Modelling root–soil interactions using three-dimensional models of root growth, architecture and function. Plant and Soil 372: 93–124.

[CIT0022] Eagles HA, Lothrop JE. 1994. Highland maize from central Mexico—its origin, characteristics, and use in breeding programs. Crop Science 34: 11–19.

[CIT0023] Erdelska O, Vidovencova Z. 1993. Development of adventitious seminal root primordia of maize during embryogenesis. Biologia Plantarum 48: 85–88.

[CIT0024] Feldman L. 1994. The maize root. In: Freeling M, Walbot V, eds. The maize handbook. New York: Springer, 29–37.

[CIT0025] Flint-Garcia SA. 2017. Kernel evolution: from teosinte to maize. In: Larkins BA, ed. Maize kernel development. Wallingford: CABI, 1–16.

[CIT0026] Flint-Garcia SA, Bodnar AL, Scott MP. 2009. Wide variability in kernel composition, seed characteristics, and zein profiles among diverse maize inbreds, landraces, and teosinte. Theoretical and Applied Genetics 119: 1129–1142. 19701625 10.1007/s00122-009-1115-1

[CIT0027] Gaines TP, Gaines ST. 1994. Soil texture effect on nitrate leaching in soil percolates. Communications in Soil Science and Plant Analysis 25: 2561–2570.

[CIT0028] Gao Y, Lynch JP. 2016. Reduced crown root number improves water acquisition under water deficit stress in maize (*Zea mays* L.). Journal of Experimental Botany 67: 4545–4557. 27401910 10.1093/jxb/erw243 PMC4973737

[CIT0029] Gaudin A, McClymont SA, Raizada MN. 2011. The nitrogen adaptation strategy of the wild teosinte ancestor of modern maize, *Zea mays* subsp. *parviglumis*. Crop Science 51: 2780–2795.

[CIT0030] Golan G, Oksenberg A, Peleg Z. 2015. Genetic evidence for differential selection of grain and embryo weight during wheat evolution under domestication. Journal of Experimental Botany 66: 5703–5711. 26019253 10.1093/jxb/erv249 PMC4566971

[CIT0031] Golan G, Hendel E, Méndez Espitia GE, Schwartz N, Peleg Z. 2018. Activation of seminal root primordia during wheat domestication reveals underlying mechanisms of plant resilience. Plant, Cell & Environment 41: 755–766. 10.1111/pce.13138 29320605

[CIT0032] Grando S, Ceccarelli S. 1995. Seminal root morphology and coleoptile length in wild (*Hordeum vulgare* ssp. *spontaneum*) and cultivated (*Hordeum vulgare* ssp. *vulgare*) barley. Euphytica 86: 73–80.

[CIT0033] Gupta SC, Radke JK, Larson WE, Shaffer, MJ. 1982. Predicting temperatures of bare- and residue-covered soils from daily maximum and minimum air temperatures 1. Soil Science Society of America Journal 46: 372–376.

[CIT0034] Hall B, Lanba A, Lynch JP. 2019. Three-dimensional analysis of biological systems via a novel laser ablation technique. Journal of Laser Applications 31: 022602.

[CIT0035] Hammer K. 1984. Das Domestikationssyndrom. Die Kulturpflanze 32: 11–34.

[CIT0036] Hastorf CA. 2009. Rio Balsas most likely region for maize domestication. Proceedings of the National Academy of Sciences of the USA 106: 4957–4958. 19321745 10.1073/pnas.0900935106 PMC2664049

[CIT0037] Ho MD, Rosas JC, Brown KM, Lynch JP. 2005. Root architectural tradeoffs for water and phosphorus acquisition. Functional Plant Biology 32: 737–748. 32689171 10.1071/FP05043

[CIT0038] Hochholdinger F, Park WJ, Sauer M, Woll K. 2004. From weeds to crops: genetic analysis of root development in cereals. Trends in Plant Science 9: 42–48. 14729218 10.1016/j.tplants.2003.11.003

[CIT0039] Huber W, Carey VJ, Gentleman R, et al. 2015. Orchestrating high-throughput genomic analysis with Bioconductor. Nature Methods 12: 115–121. 25633503 10.1038/nmeth.3252 PMC4509590

[CIT0040] Hund A, Fracheboud Y, Soldati A, Frascaroli E, Salvi S, Stamp P. 2004. QTL controlling root and shoot traits of maize seedlings under cold stress. Theoretical and Applied Genetics 109: 618–629. 15179549 10.1007/s00122-004-1665-1

[CIT0041] Hund A, Trachsel S, Stamp P. 2009. Growth of axile and lateral roots of maize: I development of a phenotying platform. Plant and Soil 325: 335–349.

[CIT0042] Hunter RB, Kannenberg LW. 1972. Effects of seed size on emergence, grain yield, and plant height in corn. Canadian Journal of Plant Science 52: 252–256.

[CIT0043] Jia X, Liu P, Lynch JP. 2018. Greater lateral root branching density in maize improves phosphorus acquisition from low phosphorus soil. Journal of Experimental Botany 69: 4961–4970. 30295904 10.1093/jxb/ery252 PMC6137997

[CIT0044] Jobbágy EG, Jackson RB. 2001. The distribution of soil nutrients with depth: global patterns and the imprint of plants. Biogeochemistry 53: 51–77.

[CIT0045] Johnson JM, Muttoni G, de Leon N, Kaeppler SM. 2016. Registration of the NyH (Ny821×H99) maize recombinant inbred mapping population. Journal of Plant Registrations 10: 101–104.

[CIT0046] Kabala C, Karczewska A, Gałka B, Cuske M, Sowiński J. 2017. Seasonal dynamics of nitrate and ammonium ion concentrations in soil solutions collected using MacroRhizon suction cups. Environmental Monitoring and Assessment 189: 304. 28567506 10.1007/s10661-017-6022-3 PMC5487726

[CIT0047] Kaeppler SM, Parke JL, Mueller SM, Senior L, Stuber C, Tracy WF. 2000. Variation among maize inbred lines and detection of quantitative trait loci for growth at low phosphorus and responsiveness to arbuscular mycorrhizal fungi. Crop Science 40: 358–364.

[CIT0048] Kiesselbach TA. 1937. Effects of age, size, and source of seed on the corn crop. Research Bulletin of the University of Nebraska College of Agriculture Agricultural Experimental Station 305: 1–16.

[CIT0049] Kiesselbach TA. 1949. The structure and reproduction of corn. Research Bulletin of the University of Nebraska College of Agriculture Agricultural Experimental Station 161: 61–84.

[CIT0050] Krasilnikov P, Gutiérrez-Castorena MD, Ahrens RJ, Cruz-Gaistardo CO, Sedov S, Solleiro-Rebolledo E. 2013. The soils of Mexico. Berlin: Springer Science & Business Media.

[CIT0051] Liben FM, Wortmann CS, Tesfaye K. 2015. Dry soil planting of maize for variable onset of rainfall in Ethiopia. Agronomy Journal 107: 1618–1625.

[CIT0052] Lynch J. 1995. Root architecture and plant productivity. Plant Physiology 109: 7–13. 12228579 10.1104/pp.109.1.7 PMC157559

[CIT0053] Lynch JP. 2013. Steep, cheap and deep: an ideotype to optimize water and N acquisition by maize root systems. Annals of Botany 112: 347–357. 23328767 10.1093/aob/mcs293 PMC3698384

[CIT0054] Lynch JP. 2019. Root phenotypes for improved nutrient capture: an underexploited opportunity for global agriculture. New Phytologist 223: 548–564. 10.1111/nph.15738 30746704

[CIT0055] Lynch JP, Brown, KM. 2001. Topsoil foraging – an architectural adaptation of plants to low phosphorus availability. Plant and Soil 237: 225–237.

[CIT0056] Matsuoka Y, Vigouroux Y, Goodman MM, Sanchez G J, Buckler E, Doebley J. 2002. A single domestication for maize shown by multilocus microsatellite genotyping. Proceedings of the National Academy of Sciences of the USA 99: 6080–6084. 11983901 10.1073/pnas.052125199 PMC122905

[CIT0057] Mayer LI, Rossini MA, Maddonni GA. 2012. Inter-plant variation of grain yield components and kernel composition of maize crops grown under contrasting nitrogen supply. Field Crops Research 125: 98–108.

[CIT0058] Meyer RS, DuVal AE, Jensen HR. 2012. Patterns and processes in crop domestication: an historical review and quantitative analysis of 203 global food crops. New Phytologist 196: 29–48. 10.1111/j.1469-8137.2012.04253.x 22889076

[CIT0059] Minow MAA, Ávila LM, Turner K, et al. 2018. Distinct gene networks modulate floral induction of autonomous maize and photoperiod-dependent teosinte. Journal of Experimental Botany 69: 2937–2952. 29688423 10.1093/jxb/ery110 PMC5972621

[CIT0060] Moegenburg SM. 1996. Sabal palmetto seed size: causes of variation, choices of predators, and consequences for seedlings. Oecologia 106: 539–543. 28307455 10.1007/BF00329713

[CIT0061] Moles AT, Ackerly DD, Webb CO, Tweddle JC, Dickie JB, Westoby M. 2005. A brief history of seed size. Science 307: 576–580. 15681384 10.1126/science.1104863

[CIT0062] Mueller SM, Messina CD, Vyn TJ. 2019. Simultaneous gains in grain yield and nitrogen efficiency over 70 years of maize genetic improvement. Scientific Reports 9: 9095. 31235885 10.1038/s41598-019-45485-5 PMC6591295

[CIT0063] de Oliveira SM, Almeida REMD, Ciampitti IA, et al 2018. Understanding N timing in corn yield and fertilizer N recovery: an insight from an isotopic labeled-N determination. PLoS One 13: e0192776. 29462178 10.1371/journal.pone.0192776 PMC5819807

[CIT0064] Paulis JW, Wall JS. 1977. Comparison of the protein compositions of selected corns and their wild relatives, teosinte and *Tripsacum*. Journal of Agricultural and Food Chemistry 25: 265–270.

[CIT0065] Paul-Victor C, Züst T, Rees M, Kliebenstein DJ, Turnbull LA. 2010. A new method for measuring relative growth rate can uncover the costs of defensive compounds in *Arabidopsis thaliana*. New Phytologist 187: 1102–1111. 10.1111/j.1469-8137.2010.03325.x 20561205

[CIT0066] Postma JA, Lynch JP. 2011 *a*. Root cortical aerenchyma enhances the growth of maize on soils with suboptimal availability of nitrogen, phosphorus, and potassium. Plant Physiology 156: 1190–1201. 21628631 10.1104/pp.111.175489 PMC3135917

[CIT0067] Postma JA, Lynch JP. 2011 *b*. Theoretical evidence for the functional benefit of root cortical aerenchyma in soils with low phosphorus availability. Annals of Botany 107: 829–841. 20971728 10.1093/aob/mcq199 PMC3077978

[CIT0068] Postma JA, Dathe A, Lynch JP. 2014. The optimal lateral root branching density for maize depends on nitrogen and phosphorus availability. Plant Physiology 166: 590–602. 24850860 10.1104/pp.113.233916 PMC4213091

[CIT0069] Postma JA, Kuppe C, Owen MR, et al. 2017. OpenSimRoot: widening the scope and application of root architectural models. New Phytologist 215: 1274–1286. 10.1111/nph.14641 PMC5575537 28653341

[CIT0070] Potocka I, Szymanowska-Pulka J. 2018. Morphological responses of plant roots to mechanical stress. Annals of Botany 122: 711–723. 29471488 10.1093/aob/mcy010 PMC6215033

[CIT0071] R Core Team. 2019. R: a language and environment for statistical computing. Vienna: R Foundation for Statistical Computing. https://www.r-project.org/.

[CIT0072] Rangarajan H, Postma JA, Lynch JP. 2018. Co-optimization of axial root phenotypes for nitrogen and phosphorus acquisition in common bean. Annals of Botany 122: 485–499. 29982363 10.1093/aob/mcy092 PMC6110351

[CIT0073] Rethemeyer J, Kramer C, Gleixner G, et al. 2005. Transformation of organic matter in agricultural soils: radiocarbon concentration versus soil depth. Geoderma 128: 94–105.

[CIT0075] Robertson BM, Waines JG, Gill BS. 1979. Genetic variability for seedling root numbers in wild and domesticated wheats 1. Crop Science 19: 843–847.

[CIT0076] Rubio G, Walk T, Ge Z, Yan X, Liao H, Lynch JP. 2001. Root gravitropism and below-ground competition among neighbouring plants: a modelling approach. Annals of Botany 88: 929–940.

[CIT0077] Saengwilai P, Tian X, Lynch JP. 2014. Low crown root number enhances nitrogen acquisition from low-nitrogen soils in maize. Plant Physiology 166: 581–589. 24706553 10.1104/pp.113.232603 PMC4213090

[CIT0078] Salvi S. 2017. An evo-devo perspective on root genetic variation in cereals. Journal of Experimental Botany 68: 351–354. 28204583 10.1093/jxb/erw505 PMC5444473

[CIT0079] Schlegel AJ, Havlin JL. 1995. Corn response to long-term nitrogen and phosphorus fertilization. Journal of Production Agriculture 8: 181–185.

[CIT0080] Schmidt JE, Bowles TM, Gaudin AC. 2016. Using ancient traits to convert soil health into crop yield: impact of selection on maize root and rhizosphere function. Frontiers in Plant Science 7: 373. 27066028 10.3389/fpls.2016.00373 PMC4811947

[CIT0081] Schneider CA, Rasband WS, Eliceiri KW. 2012. NIH Image to ImageJ: 25 years of image analysis. Nature Methods 9: 671–675. 22930834 10.1038/nmeth.2089 PMC5554542

[CIT0082] Siemens HJ. 1929. The development of secondary seminal roots in corn seedlings. Scientific Agriculture 9: 747–759.

[CIT0083] Šimunek J, Huang K, Van Genuchten MT. 1995. The SWMS_3D code for simulating water flow and solute transport in three-dimensional variably-saturated media. Riverside: U.S. Department of Agriculture Agricultural Research Service.

[CIT0084] Singh V, van Oosterom EJ, Jordan DR, Messina CD, Cooper M, Hammer GL. 2010. Morphological and architectural development of root systems in sorghum and maize. Plant and Soil 333: 287–299.

[CIT0085] Strock CF, Schneider HM, Galindo-Castañeda T, et al. 2019. Laser ablation tomography for visualization of root colonization by edaphic organisms. Journal of Experimental Botany 70: 5327–5342. 31199461 10.1093/jxb/erz271 PMC6793448

[CIT0086] Sun B, Gao Y, Lynch JP. 2018. Large crown root number improves topsoil foraging and phosphorus acquisition. Plant Physiology 177: 90–104. 29618638 10.1104/pp.18.00234 PMC5933112

[CIT0087] Tai H, Lu X, Opitz N, et al. 2016. Transcriptomic and anatomical complexity of primary, seminal, and crown roots highlight root type-specific functional diversity in maize (*Zea mays* L.). Journal of Experimental Botany 67: 1123–1135. 26628518 10.1093/jxb/erv513 PMC4753849

[CIT0088] Tai H, Opitz N, Lithio A, Lu X, Nettleton D, Hochholdinger F. 2017. Non-syntenic genes drive RTCS-dependent regulation of the embryo transcriptome during formation of seminal root primordia in maize (*Zea mays* L.). Journal of Experimental Botany 68: 403–414. 28204533 10.1093/jxb/erw422 PMC5444478

[CIT0089] Taramino G, Sauer M, Stauffer JL Jr, et al. 2007. The maize (*Zea mays* L.) RTCS gene encodes a LOB domain protein that is a key regulator of embryonic seminal and post-embryonic shoot-borne root initiation. Plant Journal 50: 649–659. 10.1111/j.1365-313X.2007.03075.x 17425722

[CIT0090] Trachsel S, Kaeppler SM, Brown KM, Lynch JP. 2013. Maize root growth angles become steeper under low N conditions. Field Crops Research 140: 18–31.

[CIT0091] Troyer AF. 1999. Background of US hybrid corn. Crop Science 39: 601–626.

[CIT0092] Troyer AF. 2004. Background of US hybrid corn II. Crop Science 44: 370–380.

[CIT0093] Turnbull LA, Philipson CD, Purves DW, et al. 2012. Plant growth rates and seed size: a re-evaluation. Ecology 93: 1283–1289. 22834369 10.1890/11-0261.1

[CIT0094] Vetsch JA, Randall GW. 2004. Corn production as affected by nitrogen application timing and tillage. Agronomy Journal 96: 502–509.

[CIT0095] Vos J, Evers JB, Buck-Sorlin GH, Andrieu B, Chelle M, de Visser PH. 2010. Functional-structural plant modelling: a new versatile tool in crop science. Journal of Experimental Botany 61: 2101–2115. 19995824 10.1093/jxb/erp345

[CIT0096] Wang J, Hu Z, Upadhyaya HD, Morris GP. 2020. Genomic signatures of seed mass adaptation to global precipitation gradients in sorghum. Heredity 124: 108–121. 31316156 10.1038/s41437-019-0249-4 PMC6906510

[CIT0097] Watson SA. 2003. Description, development, structure and composition of the corn kernel. In: White PJ, Johnson LA, eds. Corn: chemistry and technology. Saint Paul: American Association of Cereal Chemists, 69–106.

[CIT0098] White JW, Rassweiler A, Samhouri JF, Stier AC, White C. 2014. Ecologists should not use statistical significance tests to interpret simulation model results. Oikos 123: 385–388.

[CIT0099] Wickham H, Averick M, Bryan J, et al. 2019. Welcome to the Tidyverse. Journal of Open Source Software 4: 1686.

[CIT0100] Wiersum LK. 1962. Uptake of nitrogen and phosphorus in relation to soil structure and nutrient mobility. Plant and Soil 16: 62–70.

[CIT0101] Wiggans RG. 1916. The number of temporary roots in the cereals 1. Agronomy Journal 8: 31–37.

[CIT0102] Wulff RD. 1986. Seed size variation in *Desmodium paniculatum*: II. Effects on seedling growth and physiological performance. Journal of Ecology 74: 99–114.

[CIT0103] Yang HS, Janssen BH. 2000. A mono-component model of carbon mineralization with a dynamic rate constant. European Journal of Soil Science 51: 517–529.

[CIT0104] Zhan A, Lynch JP. 2015. Reduced frequency of lateral root branching improves N capture from low-N soils in maize. Journal of Experimental Botany 66: 2055–2065. 25680794 10.1093/jxb/erv007 PMC4378636

[CIT0105] Zhan A, Schneider H, Lynch JP. 2015. Reduced lateral root branching density improves drought tolerance in maize. Plant Physiology 168: 1603–1615. 26077764 10.1104/pp.15.00187 PMC4528736

[CIT0106] Zhang J, Maun MA. 1990. Seed size variation and its effects on seedling growth in *Agropyron psammophilum*. Botanical Gazette 151: 106–113.

[CIT0107] Zhu J, Mickelson SM, Kaeppler SM, Lynch JP. 2006. Detection of quantitative trait loci for seminal root traits in maize (*Zea mays* L.) seedlings grown under differential phosphorus levels. Theoretical and Applied Genetics 113: 1–10. 16783587 10.1007/s00122-006-0260-z

[CIT0108] Züst T, Joseph B, Shimizu KK, Kliebenstein DJ, Turnbull LA. 2011. Using knockout mutants to reveal the growth costs of defensive traits. Proceedings. Biological Sciences 278: 2598–2603. 21270041 10.1098/rspb.2010.2475 PMC3136827

