# Supplementary material for: Increased seminal root number associated with domestication improves nitrogen and phosphorus acquisition in maize seedlings
Source: Ann Bot. 2021 Jun 12;128(4):453–68. doi: 10.1093/aob/mcab074 (PMC8414917; doi:10.1093/aob/mcab074)
Supplement: mcab074_suppl_Supplementary_Materials [file mcab074_suppl_supplementary_materials.docx]

**Table S1.** Means, standard errors, and sample size per treatment for selected model parameters and other measurements from the maize landrace (PI 213706) and the teosinte accession (Ames 21803). Significant differences were evaluated using a Mann-Whitney test. Sample sizes vary because plants sampled close to germination had not yet developed all root classes. Seminal root diameter is not reported for teosinte because it typically did not form seminal roots.

| **Trait** | **Maize Landrace** | **Teosinte** | **n** | ***p*** |
| --- | --- | --- | --- | --- |
| Seed N Available to Seedling (μmol) | 383.9 ± 46.0 | 20.1 ± 2.9 | 4 | < 0.01 |
| Seed P Available to Seedling (μmol) | 40.8 ± 4.0 | 3.0 ± 0.1 | 3 | 0.1 |
| Seminal Root Number | 3.90 ± 0.28 | 0.25 ± 0.14 | 20 | < 0.01 |
| First Whorl Nodal Root Number | 4.42 ± 0.23 | 2.73 ± 0.14 | 12 | < 0.01 |
| Second Whorl Nodal Root Number | 4.38 ± 0.26 | 3.25 ± 0.31 | 8 | 0.02 |
| Primary Root Basal Diameter (mm) | 1.09 ± 0.03 | 0.61 ± 0.05 | 20 | < 0.01 |
| Seminal Root Basal Diameter (mm) | 0.74 ± 0.06 |  | 20 |  |
| First Whorl Nodal Root Basal Diameter (mm) | 0.84 ± 0.03 | 0.69 ± 0.02 | 12 | < 0.01 |
| Second Whorl Nodal Root Basal Diameter (mm) | 1.26 ± 0.05 | 1.00 ± 0.03 | 8 | < 0.01 |
| First Whorl Nodal Root Angle (º) | 132 ± 10 | 127 ± 14 | 3 | 1 |
| Second Whorl Nodal Root Angle (º) | 133 ± 3 | 120 ± 4 | 3 | 0.2 |
| Biomass at 25 days after planting (g) | 5.85 ± 0.36 | 1.25 ± 0.17 | 4 | 0.03 |
| Leaf area at 25 days after planting (cm^2^) | 799 ± 60 | 226 ± 21 | 4 | 0.03 |

**Table S2.** Sources of seminal root number data from other species that are shown in Fig. 10.

| **Species** | **Reference** |
| --- | --- |
| Barley | Grando and Ceccarelli, 1995 |
| Oat | Schuurman and Boer, 1970 |
| Pearl Millet | Passot *et al*., 2016 |
| Rice | Hochholdinger *et al*., 2004 |
| Rye | Pavlychenko and Harrington, 1934 |
| Sorghum | Singh *et al*., 2010 |
| Wheat | Golan *et al*., 2018 |

**Literature Cited**

Golan G, Hendel E, Méndez Espitia GE, Schwartz N, Peleg Z. 2018. Activation of seminal root primordia during wheat domestication reveals underlying mechanisms of plant resilience. *Plant, Cell & Environment* 41: 755-766.

Grando S, Ceccarelli S. 1995. Seminal root morphology and coleoptile length in wild (*Hordeum vulgare* ssp. *spontaneum*) and cultivated (*Hordeum vulgare* ssp. *vulgare*) barley. *Euphytica* 86: 73-80.

Hochholdinger F, Park WJ, Sauer M, Woll K. 2004. From weeds to crops: genetic analysis of root development in cereals. *Trends in Plant Science* 9: 42-48.

Passot S, Gnacko F, Moukouanga D, *et al*. 2016. Characterization of pearl millet root architecture and anatomy reveals three types of lateral roots. *Frontiers in Plant Science* 7: 829.

Pavlychenko TK, Harrington JB. 1934. Competitive efficiency of weeds and cereal crops. *Canadian Journal of Research* 10: 77-94.

Schuurman JJ, De Boer JJH. 1970. The developmental pattern of roots and shoots of oats under favourable conditions. *Wageningen Journal of Life Sciences* 18: 168-181.

Singh V, van Oosterom EJ, Jordan DR, Messina CD, Cooper M, Hammer GL. 2010. Morphological and architectural development of root systems in sorghum and maize. *Plant and Soil* 333: 287-299.


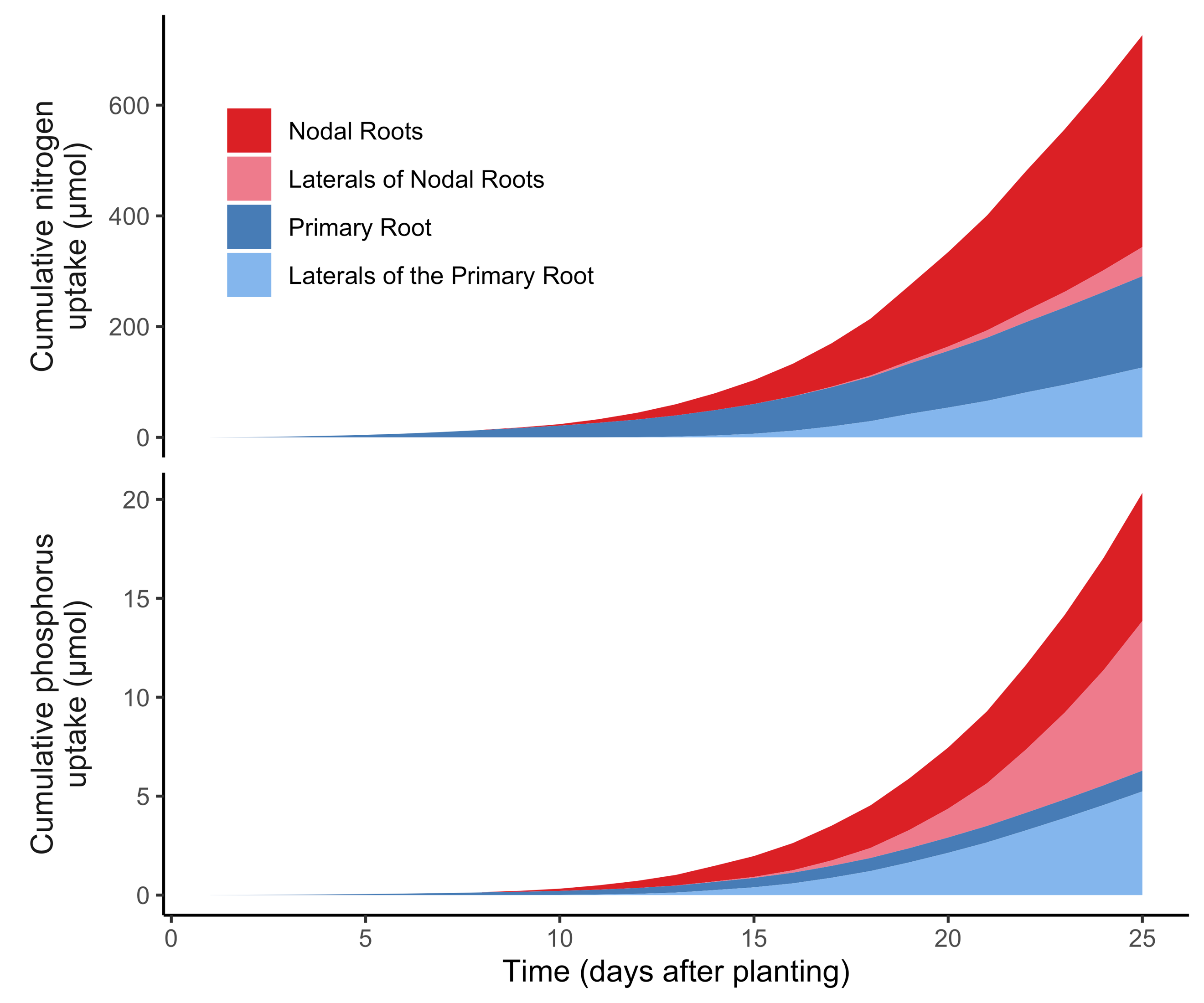


**Fig. S1.** The contribution of the primary and nodal roots to nutrient acquisition in teosinte grown in field conditions with 50 kg ha^-1^ available nitrate (top) and 2 kg ha^-1^ available phosphorus (bottom). The values presented are an average of six model replications that include stochasticity. Nodal roots emerged seven days after planting.


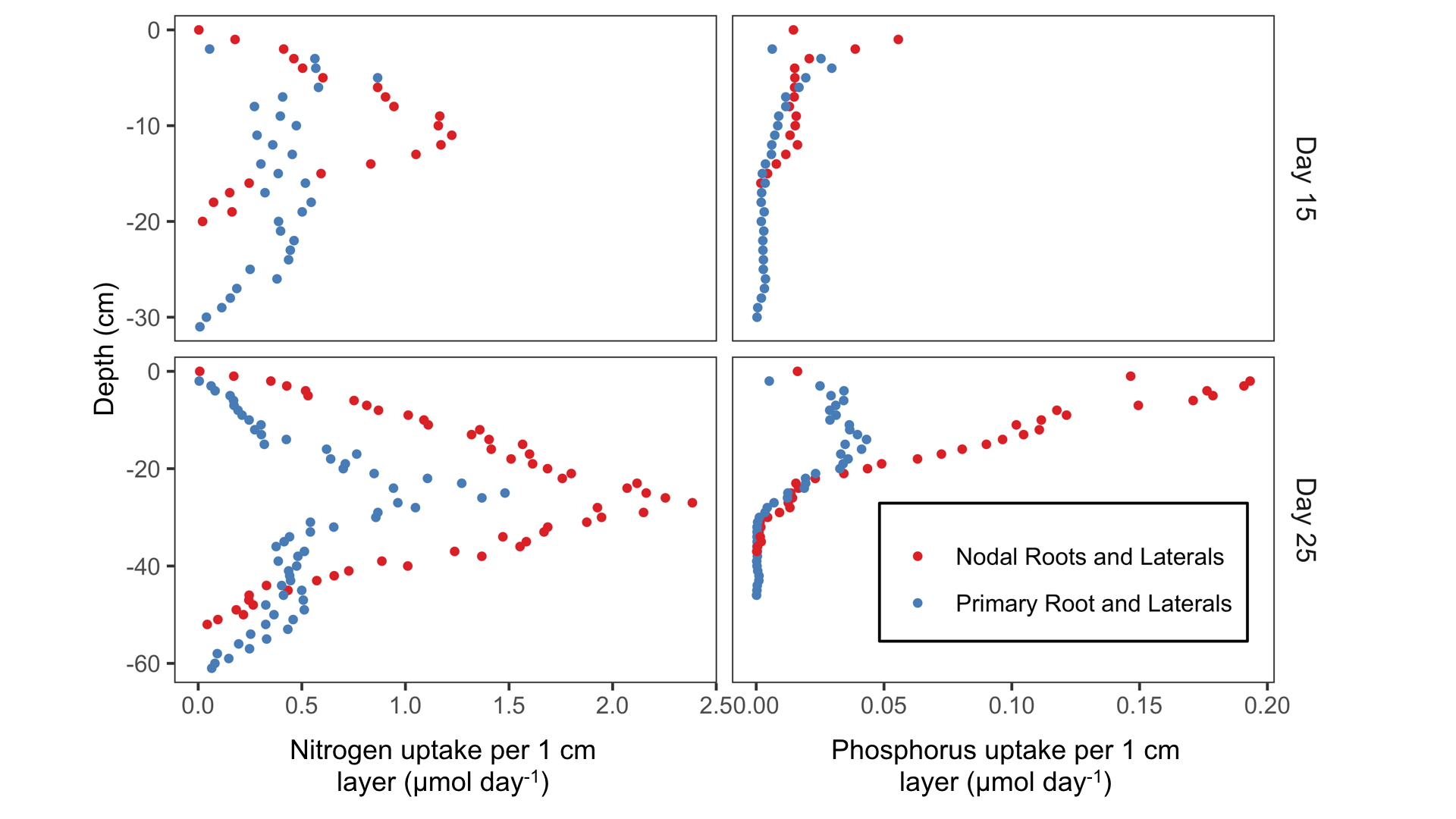


**Fig. S2.** The acquisition of nitrogen and phosphorus by teosinte at 15 and 25 days after planting. Soils with 50 kg ha^-1^ available nitrate (left) and 2 kg ha^-1^ available phosphorus (right) were used. Points represent an average of six replications.


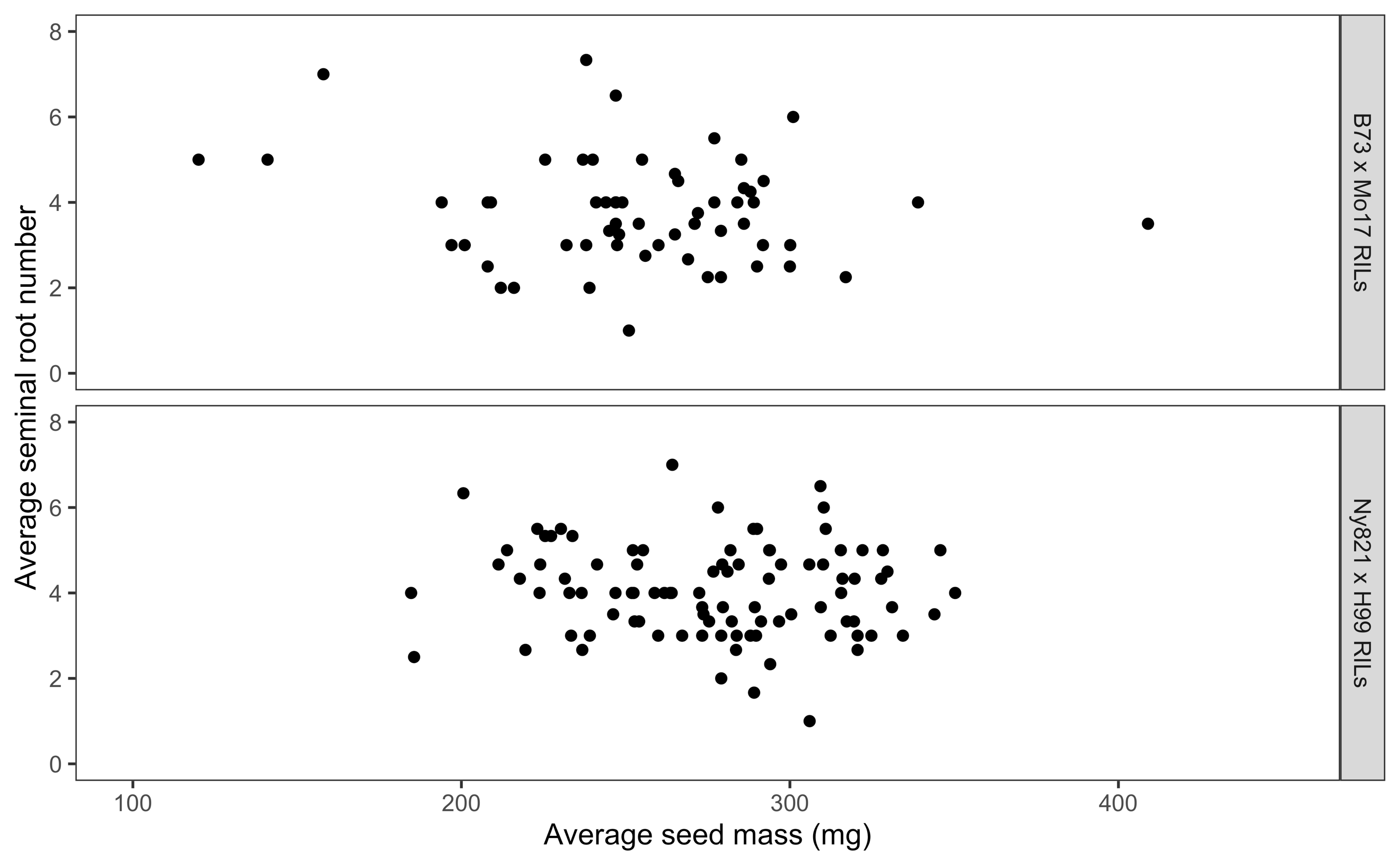


**Fig. S3.** Seminal root number does not appear to be related to seed mass in dent corn recombinant inbred lines resulting from B73 x Mo17 (Spearman’s rank correlation = -0.089) and Ny821 x H99 (Spearman’s rank correlation = -0.086).


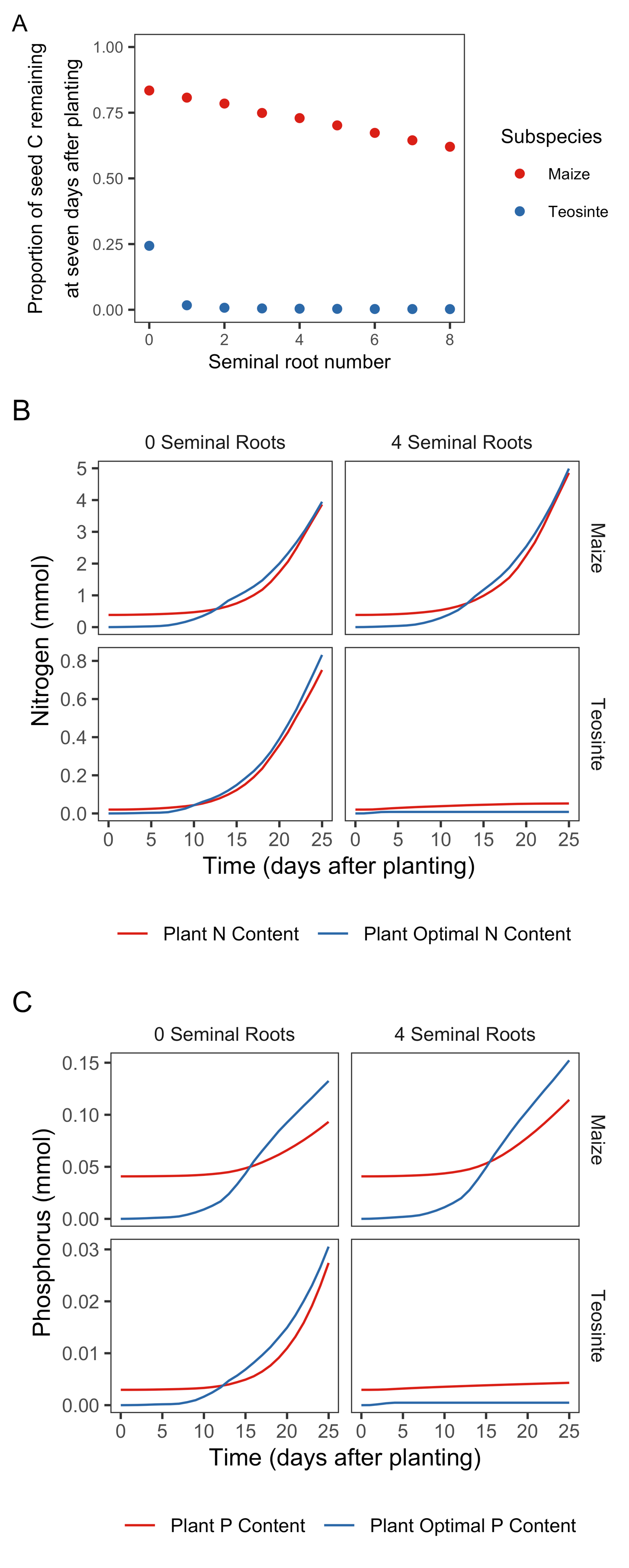


**Fig. S4.** The temporal nature of seedling carbon and nutrient stress. (A) The proportion of the starting seed carbon reserves remaining at seven days after planting for maize and teosinte. The low-N environment with 50 kg ha^-1^ available nitrate was used. (B) The timing of low-nitrogen stress onset in maize and teosinte grown in an environment with 50 kg ha^-1^ available nitrate. (C) The timing of low-phosphorus stress onset in an environment with 2 kg ha^-1^ available phosphorus.


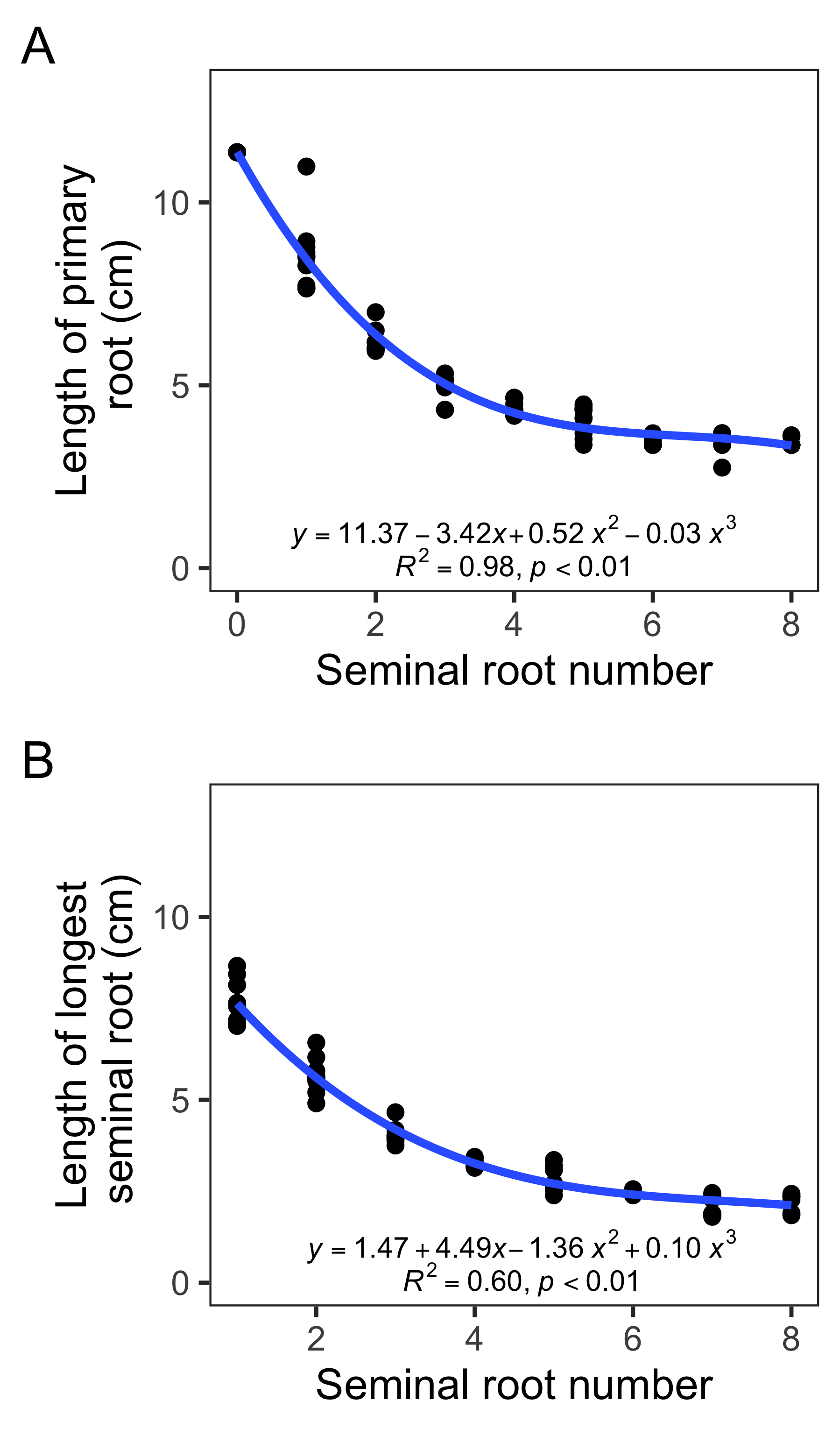


**Fig. S5.** The impact of seminal root number on the length of the primary root and longest seminal root in teosinte at seven days after planting. This tradeoff exists due to the limited seed carbohydrate reserves of teosinte. Points are values from individual model runs.
